# A Wireless, Passive Multimodal Wearable Sensor With Decoupled Sensing Capabilities

**DOI:** 10.1002/adma.74293

**Published:** 2026-07-30

**Authors:** Wenjiang Han, Heng Xiao, Tianshuang Wang, Xiaoran Ding, Liupeng Zhao, Seokjoo Cho, Peng Sun, Inkyu Park, Geyu Lu

**Affiliations:** ^1^ State Key Laboratory of Integrated Optoelectronics (JLU Region) College of Electronic Science and Engineering Jilin University Changchun China; ^2^ International Center of Future Science Jilin University Changchun China; ^3^ Department of Mechanical Engineering Korea Advanced Institute of Science and Technology (KAIST) Daejeon Republic of Korea

**Keywords:** electronic skin (e‐skin), frequency‐division multiplexing, LC resonator array, multimodal decoupled sensing, wireless passive wearable sensor

## Abstract

Developing electronic skin (e‐skin) that perceives multi‐stimuli and even exceeds human skin sensing capacity remains a major challenge. Yet existing technologies focused mainly on tactile sensing suffer from poor superimposed multi‐stimuli discrimination, external power dependence, and wired transmission modes. Here, we report an integrated wireless passive wearable sensor based on frequency‐division inductance‐capacitance (LC) resonator array, capable of simultaneously detecting superimposed multi‐stimuli including pressure, odor and humidity, along with decoupling. The system's performance is designed and validated using three‐dimensional full‐wave electromagnetic simulations. Notably, pressure‐sensing with an ultrafast response/recovery (∼5/6 ms) and high sensitivity (6.15 MHz·kPa^−1^) is enabled by a gradient‐modulus trilayer hydrogel incorporating a micro‐pyramidal patterned top‐layer. Furthermore, the sensor demonstrates trace‐level (200 ppb) NO_2_ detection without interference from humidity or pressure and exhibits high humidity sensitivity across a wide humidity range (2%–98% RH). Demonstration of this sensor as e‐skin reveals capabilities surpassing previous devices, enabling wireless passive and decoupled detection of small applied mechanical pressure, trace‐level NO_2_, and ambient humidity under complex stimuli conditions, showing high selectivity and minimal cross‐sensitivity. The proposed system introduces a transformative approach, unlocking substantial benefits for a variety of wearable applications.

## Graphical abstract

A wireless passive multimodal electronic skin is presented for the simultaneous perception of pressure, humidity, and odor through a frequency‐divided LC resonator array. The decoupled sensing architecture suppresses cross‐interference and enables independent signal readout under superimposed stimuli while maintaining high stability. The platform surpasses previously reported multimodal wearable sensors, offering a powerful strategy for next‐generation intelligent wearable sensing.

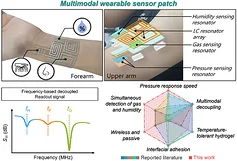

## 1 Introduction

Recent advances in wearable sensors have enabled devices capable of continuously and simultaneously monitoring physiological, environmental or motion‐related parameters while being comfortably affixed to the body [1, 2], driving progress in healthcare, fitness, safety, and even entertainment [3, 4, 5, 6]. Their key attributes are portability, energy efficiency, adaptability, and multi‐stimuli simultaneous measurement, making them versatile tools applicable across numerous fields [6, 7, 8]. However, most wearable sensors still rely on wired communication, which compromises system autonomy, introduces parasitic capacitance crosstalk, limits flexibility and mobility, increases the risk of tangling or damage, and adds maintenance challenges [9, 10, 11].

To address this issue, wearable sensors have evolved toward wireless communication, achieved through magnetic wave transmission for information exchange, such as Bluetooth, and Wi‐Fi technologies, while simultaneously integrating stimulus signal acquisition and data transmission [12, 13, 14]. However, conventional wireless communication systems require an active power source, such as battery or generator, inevitably leading to shorter operational lifespans, larger sizes, and higher maintenance needs [15]. Currently, the design of wireless passive sensors, such as radio frequency identification devices (RFID), and near field communication (NFC) sensing systems, is rapidly emerging as an alternative to conventional wireless active‐powered devices as they can be engineered to consume less power, possess smaller sizes, enhance wearable sensors’ flexibility and mobility, and reduce the need for user intervention [16, 17]. However, conventional RFID‐/NFC‐based wearable sensors generally require microchip integration, which not only increases manufacturing complexity but also introduces rigid components (e.g., resistor and capacitance) that compromise device flexibility [16, 18].

Alternatively, cost‐effective LC resonator operating without a power source detects resonance frequency shift induced by physical or chemical stimuli, eliminating the need for microchips, reducing manufacturing complexity, enhancing flexibility and scalability, consuming ultra‐low power, and enabling long‐range and real‐time monitoring [19, 20]. The resonance frequency of LC resonator can be precisely tuned by adjusting the number of turns in the inductance coil or modifying the capacitance value, enabling the design of interconnected multiple resonators rather than independent and discrete units [20, 21]. This integration enhances system performance and reliability. Additionally, discrete frequency allocation assigning each resonator a unique operating frequency to differentiate signals in the frequency domain enables the decoupling of multi‐stimuli, allowing for their simultaneous detection [21, 22]. Although LC‐based wearable sensors have advanced rapidly, most existing studies still focus on single‐modality sensing [19, 23, 24]. Reported multimodal LC sensors typically rely on a single sensing material or a single resonant circuit architecture to respond to multiple stimuli [25, 26], often requiring algorithm‐assisted decoupling [23, 27] and increasing signal processing complexity. In addition, current LC‐based multimodal systems mainly target mechanical, temperature, and humidity parameters, while integration of gas sensing remains limited [28, 29, 30, 31, 32, 33]. Their sensing performance is further constrained by insufficiently fast tactile response, narrow humidity detection ranges (25–70% RH) [29, 34], relatively high gas detection limits (e.g., 500 ppb) [35], and limited validation of anti‐interference ability under coupled humidity and mechanical perturbations. Since gas and humidity responses are prone to cross‐interference [19], and mechanical deformation can further perturb capacitance and resonance frequency stability [27], algorithm‐free and interference‐resistant integration of tactile, olfactory, and humidity sensing on a single flexible wireless passive platform remains a critical challenge for next‐generation e‐skin.

**FIGURE 1:**
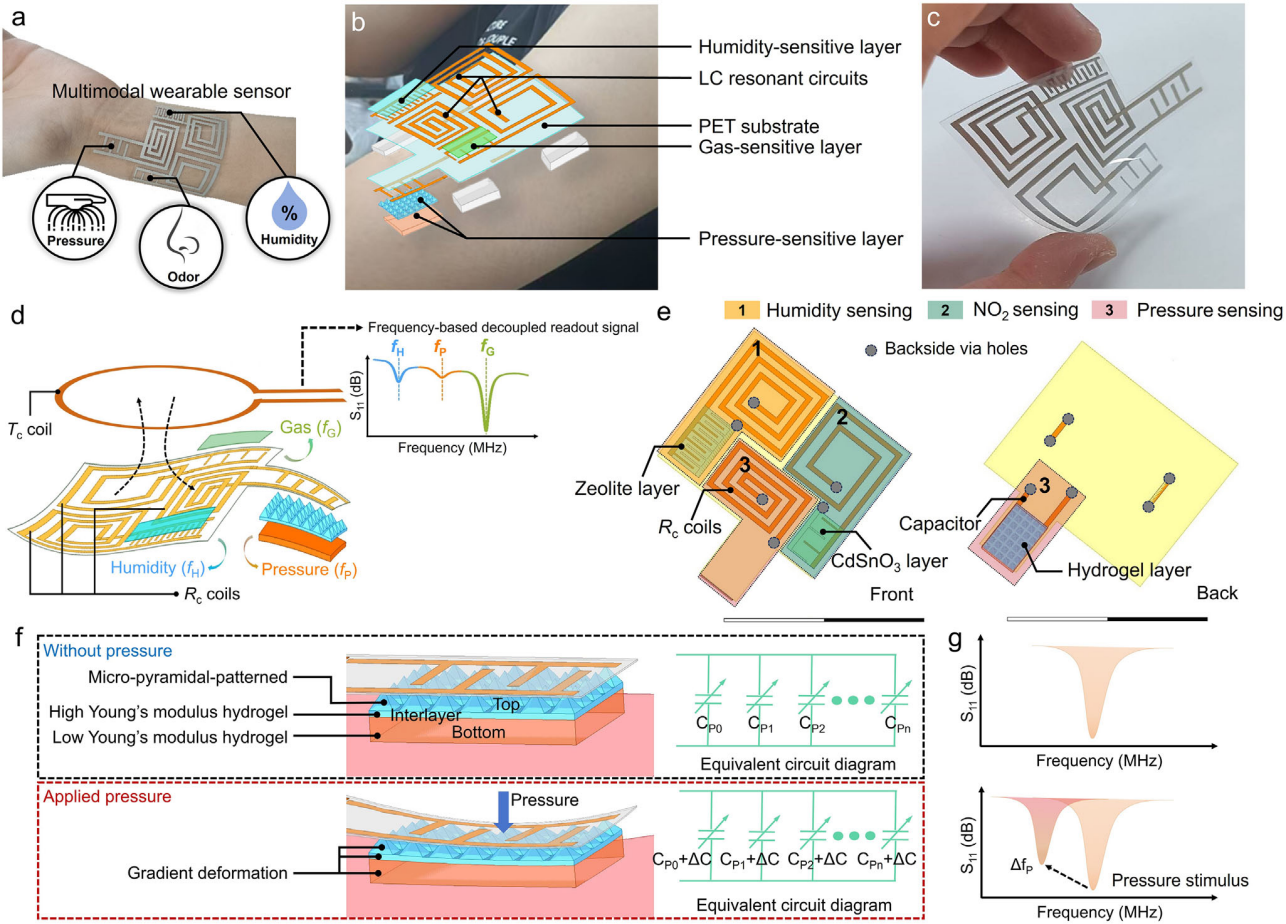
Wireless passive wearable sensor as an e‐skin for multimodal perception. (a) Schematic of multi‐stimuli sensing system integrating pressure, odor, and humidity modalities. The flexible resonator array is fabricated on a polyethylene terephthalate (PET) substrate with patterned silver (Ag) circuitry, offering skin‐conformable mechanical flexibility. (b) Multilayered architecture of the IWP wearable sensor patch. (c) Photograph of the LC resonator array composed of interdigitated electrodes and spiral inductance coils (*R*_c_). (d) Multiplexed readout of pressure, humidity, and NO_2_ gas signals for decoding multi‐stimuli. (e) HFSS simulation layout for LC resonator structures. Scale bar, 60 mm. (f) Detailed structure and sensing mechanism of the pressure‐sensing unit based on trilayer hydrogel with gradient modulus, and micro‐pyramidal patterned top‐layer, along with the corresponding equivalent circuits. (g) Resonance frequency‐based pressure response characteristic.

Here, we present an integrated wireless passive (IWP) wearable multimodal sensor based on frequency‐division LC resonator array, comprising three distinct LC resonators units—dedicated to pressure, gas and humidity sensations—each formed by a capacitive sensing element connected in series with an inductance coil (Figure 1a). Each resonator unit is designed using high‐frequency structure simulator (HFSS) to operate at a distinct resonance frequency with frequency intervals exceeding 93 MHz, enabling multi‐band wireless readout that minimizes cross‐interference. This design allows for simultaneous, decoupled detection of superimposed multi‐stimuli with high signal integrity. Additionally, sensing materials are independently tailored for each unit to further suppress cross‐interference. The cantilever‐type pressure‐sensing unit is constructed from a gradient‐modulus trilayer hydrogel with micro‐pyramidal patterned top layer, exhibiting a fast response/recovery time of 5/6 ms, also offering long‐term antifreezing and anti‐dehydration stability across a broad temperature range (‐20–50°C). The humidity‐sensing unit based on zeolites exhibits high sensitivity (0.572 MHz∙%RH^−1^) over a wide humidity range of 2%–98% RH and long‐term stability over 18 days, while remaining unaffected by pressure and gas interference. The gas‐sensing unit employs CdSnO_3_, enabling highly selective and humidity‐resistant detection of trace‐level NO_2_ (200 ppb). Importantly, the sensing mechanism of LC resonators governed by dielectric constants (*ε*', *ε*′′) and loss tangent (tan *δ*) is validated via in situ dielectric probe measurement. As proof of concept, the IWP wearable sensor demonstrates robust multi‐stimuli detection and precise signal discrimination via frequency‐based decoupling, underscoring its potential as an e‐skin platform.

## 2 Result and Discussion

### 2.1 Materials and Device Design Layouts

The exploded‐view schematic illustration highlights the sensor architecture (Figure 1b), comprising polyethylene terephthalate (PET) substrate and three independent LC resonant circuits, each integrated with a dedicated stimuli‐sensitive layer. A photograph of the resonator array patch demonstrates its flexibility (Figure 1c). The wireless multiplexed sensing system operates via magnetic inductive coupling between an external near‐field radio‐frequency (RF) transmission coil (*T*_c_) and a matched receiver coil (*R*_c_) embedded in the sensor (Figure 1d). The RF readout network employs a single‐port planar *T*_c_ connected to a portable vector network analyzer (VNA) for electromagnetic excitation and return loss (*S*_11_) measurement. The sensor adopts a modular architecture, functionally divided into pressure‐, humidity‐, and gas‐sensing units. Each unit is electromagnetically coupled in parallel to *T*_c_, with stimulus‐induced capacitance changes shifting the resonance frequency to generate the sensing output.

Key innovations reported here include the optimized LC resonant circuit structures (Figure 1e), tailored sensing layers design (Figures S1–S4, and the Supporting Methods), and a trilayer hydrogel for pressure sensing (Figure 1f). The first improvement involves multimodal signals decoupling through the frequency‐division resonator array. Each LC resonator unit is engineered to operate at a distinct frequency by tuning the receiver coil's (*R*_c_) number of turns and dimensions. Additionally, the use of oppositely wound spiral coils effectively mitigates mutual inductance and suppresses inter‐device cross‐coupling [36]. As a result, distinct resonance frequencies corresponding to pressure (denoted as *f*_P_), humidity (denoted as *f*_H_), and NO_2_ gas (denoted as *f*_G_) enable simultaneous, multiplexed readout through a single RF frequency scan (top of Figure 1d). Specifically, the front‐side PET substrate hosts the *R*_c_ components of the pressure‐, humidity‐, and gas‐sensing LC resonators, allowing synchronized and multiplexed coupling with one *T*_c_.

The second improvement features three dedicated capacitive sensing layers that function as the capacitive components in their respective LC resonant circuits. Specifically, CdSnO_3_ and zeolite serve as the gas‐ and humidity‐sensing layers, respectively, positioned on the sensor's upper side (left of Figure 1e). To prevent cross‐coupling between sensing modalities, particularly pressure‐induced artifacts in the humidity‐/gas‐sensing units, a hydrogel‐based capacitive sensing layer configured in a cantilever geometry is isolated on the sensor's backside (right of Figure 1e). Meanwhile, the electrical interconnection between the front‐side *R*_c_ and back‐side capacitive component of the pressure‐sensing resonator is achieved through backside via holes.

The third improvement includes two aspects associated with the trilayer hydrogel dielectric layer (Figure 1f): a trilayer (bottom–interlayer–top) structure with a gradient in Young's modulus, and a micro‐patterned surface. The bottom layer, composed of a low Young's modulus adhesive hydrogel (LYAH), interfaces with the skin to capture subtle mechanical stimuli. The intermediate layer, made of high Young's modulus hydrogel (HYH), extends the pressure detection range, as described in detail in the next section. The top layer of micro‐pyramidal‐patterned HYH positioned adjacent to the interdigitated electrodes of the capacitive section enhances pressure sensitivity. Each micro‐pyramid functions as a discrete dielectric element, together forming a parallel array of capacitive components. Mechanical deformation of this structure modulates the capacitance of the LC resonator, resulting in a measurable shift in resonance frequency (Figure 1g).

### 2.2 Design Principle and Characterizations of Pressure‐Sensing Hydrogels

**FIGURE 2:**
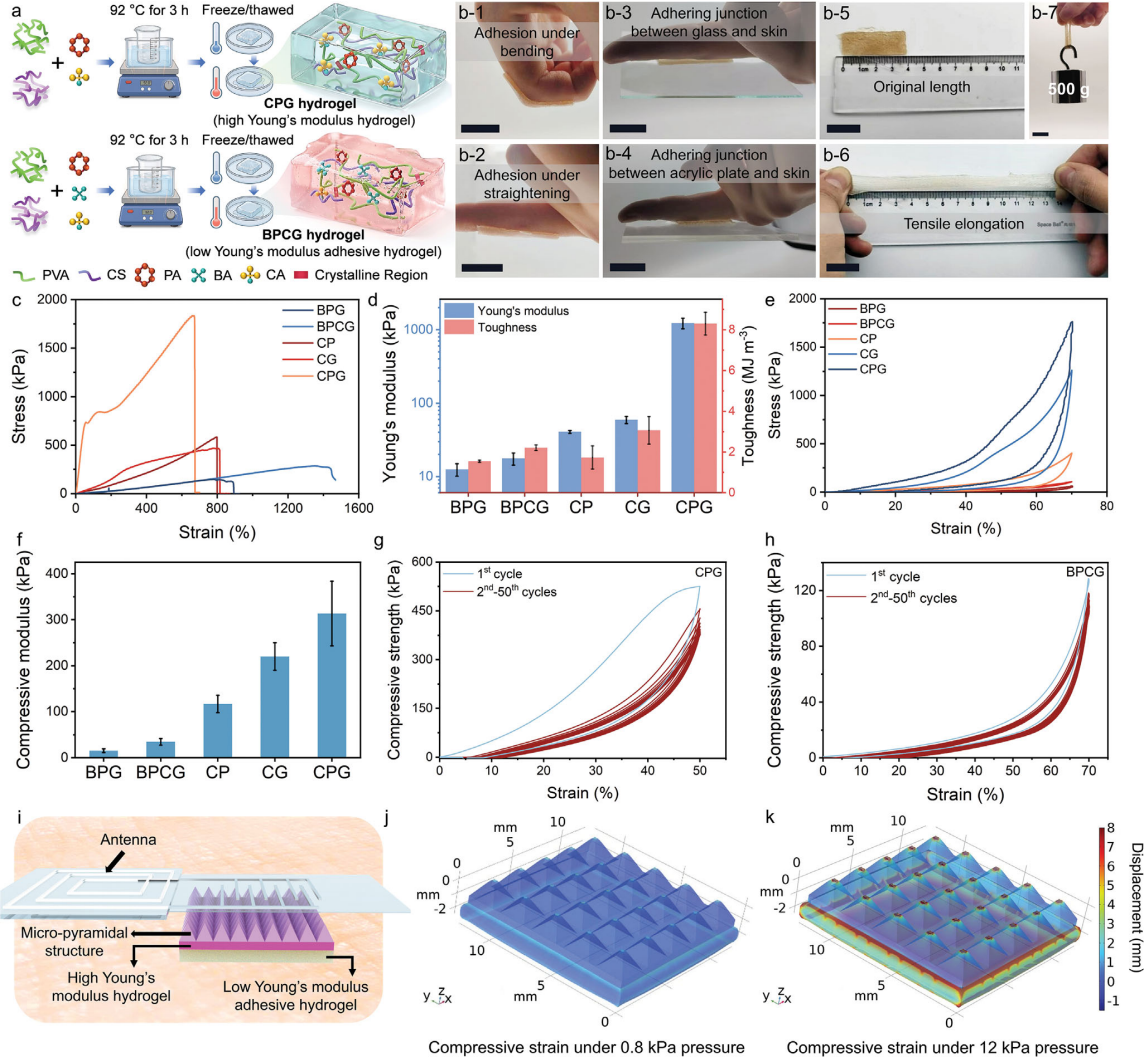
Design of high‐performance hydrogel for pressure‐sensing resonator. (a) Schematic illustration of the preparation process and cross‐linking structure of PVA‐CS hydrogel, where the Young's modulus can be tuned by incorporating different natural organic acids, such as phytic acid (PA), citric acid (CA), and boric acid (BA). (b) Photographs of the **BPCG** organohydrogel stuck on various surfaces—including knuckle skin (b‐1, b‐2), glass (b‐3), and acrylic panels (b‐4)—as well as demonstrating its ability to withstand large tensile deformations (b‐5, b‐6) and support a 500 g tensile load (b‐7). Scale bar, 20 mm. (c and d) Tensile stress–strain curves (c) and the corresponding Young's modulus and toughness values (d) of **BPG**, **BPCG**, **CP**, **CG**, and **CPG** hydrogels. (e and f) Compressive stress‐strain curves (e) and the corresponding compressive modulus values (f) of **BPG**, **BPCG**, **CP**, **CG**, and **CPG** hydrogels. (g and h) Cyclic loading–unloading compressive testing for 50 cycles of **CPG** (g) and **BPCG** (h) organohydrogels. (i) Structural diagram of the pressure‐sensing resonator unit. (j and k) Finite‐element analysis (FEA) results of strain distribution in the organohydrogel‐based pressure‐sensing layer under applied pressure.

To meet the requirement for flexibility and biocompatibility, polyvinyl alcohol (PVA) and chitosan (CS) are used as the polymeric framework, with a glycerol‐water binary solvent system. The precursors are treated in a water bath and subjected to freeze‐thaw cycling to form PVA‐CS dual‐network organohydrogels. Fourier transform infrared (FT‐IR) spectroscopy confirms cross‐linking interactions between PVA and CS (Figure S4) [37, 38]. To evaluate the influence of cross‐linking components and solvent composition, five hydrogel formulations are designed and categorized into two functional types: low‐modulus adhesive hydrogels (**BPG** (boric acid + phytic acid + glycerol); **BPCG** (boric acid + phytic acid + citric acid + glycerol)), and high‐modulus hydrogels (**CP** (citric acid + phytic acid); **CG** (citric acid + glycerol); **CPG** (citric acid + phytic acid + glycerol)). Notably, **CP** is formulated without glycerol to serve as a control for evaluating glycerol's role in water retention and flexibility (Figure S5). Detailed compositions and comparative profiles are summarized in Supporting Discussions 2.1, and Tables S1 and S2 (Supporting Information). Among these, **BPCG** exhibits strong adhesion, excellent stretchability, and low Young's modulus, thereby being suitable as a skin‐interfacing layer (Figure 2a,b). In contrast, **BPG** delivers good adhesion but limited mechanical robustness, while **CPG** provides a high modulus but lacks adhesive property. **BPCG** achieves an optimal balance, enabling conformal contact with skin or glass and supporting tensile loads up to 500 g (Figure 2b). To quantitatively evaluate its adhesion performance, standard 180° peeling tests were conducted. The results demonstrate that the BPCG hydrogel achieves robust interfacial toughness values of approximately 60.9, 26.5, and 34.6 J/m^2^ on acrylic plates, glass substrates, and pigskin, respectively (Figure S6). Incorporation of glycerol in the synthesis of hydrogels significantly enhances their environmental robustness, surpassing previously reported materials [39]. The **BPCG** and **CPG** hydrogels retain over 88% and 77% of their original weight after exposure to elevated temperatures and maintain flexibility down to ‐20 °C (Figures S7 and S8). Thermogravimetric analysis (TGA) and differential scanning calorimetry (DSC) confirm suppressed ice crystallization and reduced evaporation rates (Figures S9 and S10) [40, 41]. Long‐term ambient storage tests further demonstrate excellent water retention (Figure S11).

To tune Young's modulus, the introduction of citric acid markedly enhances the mechanical properties of the organohydrogel. Under uniaxial tensile testing, the soft **BPCG** demonstrates skin‐like compliance with a Young's modulus of 17.6 kPa matching that of human skin, and exhibits ∼160% greater strain capability than **BPG**, resulting in improved toughness of 2.2 MJ·m^−3^ (Figure 2c,d) [42]. The tough **CPG** exhibits superior environmental stability alongside remarkable mechanical enhancement, with a 30.4‐fold increase in Young's modulus (1233.6 kPa) and a 4.9‐fold improvement in toughness (8.3 MJ·m^−3^) compared to the glycerol‐free **CP** hydrogel (Young's modulus: 40.6 kPa; toughness: 1.7 MJ·m^−3^). This enhancement is attributed to glycerol's abundant hydroxyl groups, which act as additional hydrogen bond donors to reinforce the cross‐linking network [43].

To elucidate the role of phytic acid in the **CPG** organohydrogel, a control sample (**CG**) is prepared using only citric acid and glycerol. The markedly lower Young's modulus of **CG** (59.4 kPa) suggests that the synergistic cross‐linking among phytic acid, citric acid, and glycerol underpins the exceptional mechanical strength of **CPG** (Figure 2c,d). Compression tests further highlighted their contrasting mechanical properties: BPCG was soft and compliant, with a compressive modulus of 34.7 kPa, whereas CPG was stiff and load bearing, with a modulus of 313.3 kPa and a compressive stress of 1763.9 kPa at 70% strain (Figure 2e,f). This substantial enhancement is attributed to citric acid's ability to increase cross‐linking density via abundant hydrogen bonding sites and complex topology, surpassing the conventional boric acid cross‐linker. Cyclic compression tests reveal minimal hysteresis attenuation after the initial cycle and stable performance over 50 cycles (Figure 2g,h), indicating excellent fatigue resistance and a steady‐state energy dissipation behavior, primarily resulting from the reversible reorganization of the dynamic cross‐linked network [44].

Based on the above analysis, **BPCG** (LYAH) serves as the bottom layer to ensure conformal and stable contact with the skin, while **CPG** (HYH) is used to fabricate both the micro‐pyramid patterned top layer and the compressive supporting interlayer (Figures S12 and S13). Collectively, the trilayer hydrogel interfaces with the backside interdigitated electrodes to form the capacitance, which combined with the front *R*_c_ constitutes the pressure‐sensing resonator unit (Figure 2i). A finite‐element analysis hyperelastic model of the trilayer hydrogel is established to elucidate the pressure‐sensing mechanism (Figures 2j,k and S14). At low pressures (< 4 kPa), minimal deformation occurs in both the micro‐pyramidal top layer and the bottom adhesive layer. As pressure increases, the bottom layer exhibits greater displacement, while stress concentrates in the top and interlayer regions, indicating that the **CPG** effectively withstands higher loads (Figures S14–S16). The contact area between the micro‐pyramid tips and the PET substrate increases, accompanied by reduced air volume in the gaps (Figure S17). The enlarged contact area lowers the capacitance's impedance, while the reduced air volume alters the dielectric environment of the pressure‐sensing LC resonator unit. This dual‐mode modulation, leveraging changes in both conductivity and dielectric constant, greatly enhances pressure sensitivity. Thus, the hierarchical deformation of the trilayer hydrogel, comprising a soft adhesive bottom layer, a rigid supporting interlayer, and a micro‐pyramid patterned top layer, enables both ultrahigh sensitivity and a broad pressure detection range.

### 2.3 Frequency‐Decoupled Operational Designs

**FIGURE 3:**
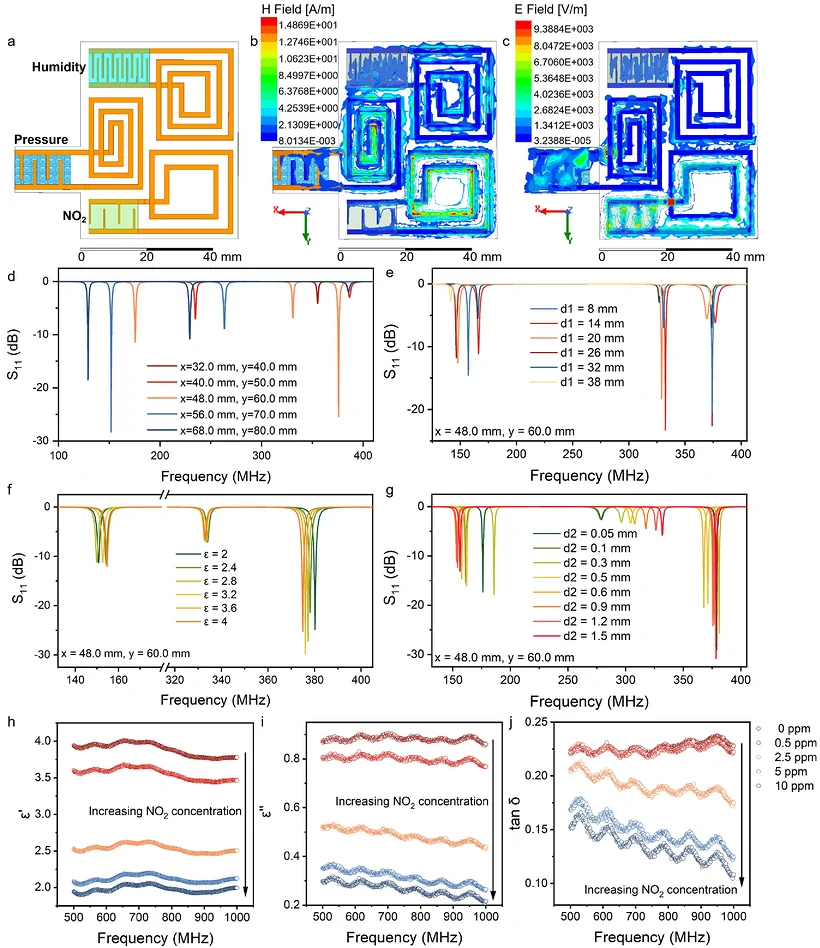
Electromagnetic simulations and operational mechanism analyses. (a–c) Schematic illustration for the IWP sensor layout (a), and modeling results for the magnetic inductive coupling between *T*_c_ and *R*_c_: magnetic field (b), and electric field (c) distributions. (d–g) Modeling results for the *S*_11_ as a function of device size (d), distance from *T*_c_ to *R*_c_ (*d*1) (e), dielectric constant variation at the gas‐ and humidity‐sensing ports (f), and deformation at the pressure‐sensing port (*d*2) (g). (h–j) in situ dielectric permittivity measurement of the dielectric constant (h), dielectric loss (i), and loss angle tangent (j) of the gas‐sensing layer in LC resonator under varying NO_2_ concentrations (0–10 ppm).

The electromagnetic performance of the *T*_c_ and the wireless passive resonator array coated with sensing layers were modeled using ANSYS HFSS to design an IWP sensor with high quality factor (*Q* factor), stable resonance frequency, and multi‐band communication capability (Figure 3a). Simulations show strong magnetic fields concentrated in the resonator's *R*_c_ and intense electric fields localized in the capacitance (Figure 3b). Thus, sensing layers are deposited on the capacitive components, where the strong electric field is highly sensitive to dielectric changes, enabling resonance frequency tuning through capacitance modulation [45]. The system consists of a *T*_c_ electromagnetically coupled to three parallel LC resonators. Each resonator comprises a planar spiral inductor (*L*_X_), parasitic resistance (*R*_X_), and variable capacitance (*C*_X_), ensuring the frequency‐based decoupling (Figure S18). The resonance frequency *f*_X_ relates to the sensor's capacitance and inductance as the following equation [46]:

(1) $$f_{X}=\frac{1}{2\pi \sqrt{L_{X}C_{X}}}\;$$

Electromagnetic simulations using a 60 mm‐diameter copper as *T*_c_ guided the design of a miniaturized resonator array patch with a high *Q* factor. Varying the sensor patch dimensions (length (*y*): ranging from 40.0 to 80.0 mm, width (*x*) from 32.0 to 64.0 mm) could impact the *Q* factor, *S*_11_, and resonance frequencies (Figures 3d and S19). When the device is small, only a single reflection peak from one unit is detectable, while the other two remain obscured. As the size increases, two additional peaks emerge, though their *Q* factors remain low. At the optimal size of 48.0 × 60.0 mm^2^ (width × length), three well‐defined resonance peaks emerge, demonstrating significantly improved *Q* factors of 64, 158.74, and 125.36 (left to right), indicating optimized performance. The higher *Q* factor can amplify frequency shifts induced by dielectric changes in capacitance, thereby enhancing sensitivity [47]. Conversely, when the device size exceeds this optimum, the two lower‐frequency resonance peaks intensify while the highest‐frequency peak weakens markedly, due to incomplete magnetic coupling between the oversized resonator array and the *T*_c_. The Q factor is defined as follows, Δ*f*_FWHM_ is the full width at half maximum (FWHM) [48].

(2) $$Q=\frac{1}{R_{X}}\;\sqrt{\frac{L_{X}}{C_{X}}}\approx \;{\frac{f_{X}}{\Delta f}}_{\mathrm{FWHM}}$$

Basically, loading the sensing layer as a dielectric onto the capacitor reduces the reflection coefficient and shifts the LC resonator's resonance frequency. To evaluate these effects, electromagnetic simulations were performed, revealing a uniform low‐frequency shift in all resonance peaks after sensing layer deposition. This behavior aligns with electromagnetic coupling tests conducted on the IWP sensor before and after loading (Figures S19 and S20, and Equation 1), confirming an increase in the resonant circuits’ effective capacitance. Further simulations investigated the influence of the distance (*d*1) between the *T*_c_ and *R*_c_ on the reflection coefficient. The results identify a critical maximum magnetic inductive coupling distance of 26 mm (Figure 3e), beyond which coupling strength diminishes, as evidenced by a decline in *S*_11_. Even at the extended reading distance of 26 mm, the system maintains high‐quality long‐range wireless transmission, supported by a consistently high *Q* factor (Table S3).

The IWP sensor's response mechanism relies on resonance frequency shifts, primarily governed by variations in the dielectric constant (*ε*) of the sensing layer upon stimulus contact. Electromagnetic simulations model gas (or water) molecules binding to the sensing layer, where increasing concentrations raise *ε*, leading to a progressive decrease in the resonance frequency and *S*_11_ of the gas‐ or humidity‐sensing resonator unit (the rightmost peak) (Figure 3f), due to modulated propagation and reflection of incident electromagnetic waves within the resonator. For the pressure‐sensing resonator, simulations model the mechanical deformation of the hydrogel layer, where the distance (*d*2) between the micro‐pyramidal top layer and capacitor electrodes represents the compression level. As *d*2 decreases with increased compression, the resonance frequency (*f*_P_) gradually shifts lower (Figure 3g), due to progressive replacement of air by hydrogel in the sensing region, increasing capacitance (*C*_P_) (Figure S17, and Equation 1). Concurrently, the enlarged contact area raises conductivity in the capacitive section, reducing *Q* factor and *S*_11_ (Equations 2 and 3, where ω is angular frequency).

(3) $$\varepsilon^{\prime \prime }\propto \;\frac{\sigma }{\omega }$$

(4) $$\tan\delta =\frac{\varepsilon^{\prime \prime }}{\varepsilon^{\prime }}$$

We further developed an in situ dielectric permittivity measurement to monitor the dielectric parameters (*ε’*, *ε″*, and dielectric loss tangent tan *δ*) of the sensing layer under varying NO_2_ concentrations (Figures 3h–j and S22). *ε’* decreases, as increasing NO_2_ concentration, consistent with simulation results. This reduction is attributed to NO_2_ adsorption, which withdraws electrons from the sensing layer, thereby decreasing carrier concentration and electrical conductivity (*σ*) [49]. The reduced conductivity leads to concurrent decreases in *ε*″ and tan *δ*, as described by Equations 3 and 4, in agreement with experimental observations.

### 2.4 Multimodal Sensing Capabilities of the IWP Sensor

**FIGURE 4:**
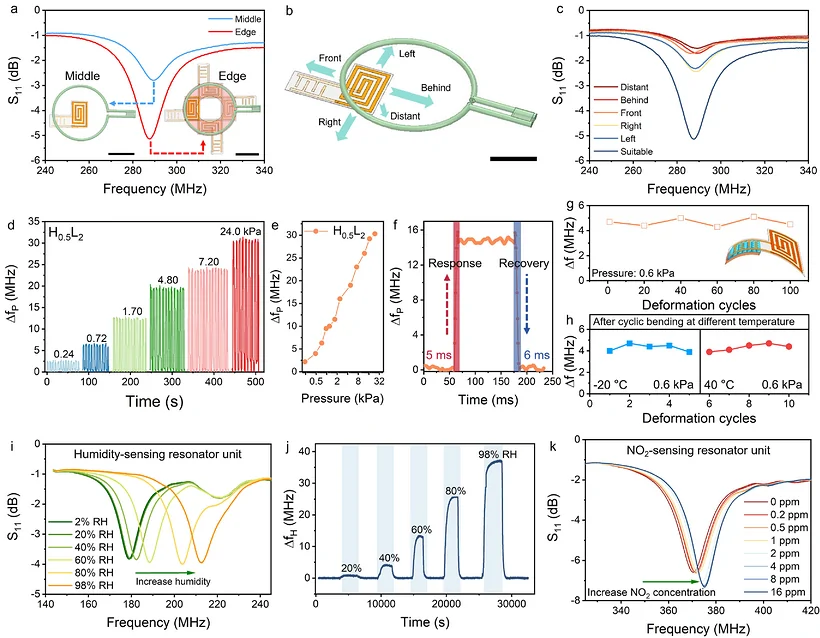
Multimodal sensing capability of the IWP sensor for multi‐stimuli detection including pressure, humidity, and NO_2_. (a) Schematic illustration of the LC resonator unit positioned at both the center and periphery of the *T*_c_, alongside their corresponding simulated *S*_11_ versus resonance frequency response characteristics. Scale bar, 30 mm. (b and c) Resonator's misalignment tolerance was evaluated under 20–30 mm displacements (lateral, longitudinal, and diagonal) relative to the readout coil. Scale bar, 20 mm. (d–h) Performance characterization of pressure‐sensing resonator unit: Resonance frequency shifts of the H_0.5_L_2_ resonator under applied pressures ranging from 0.24 to 24.00 kPa (d); Pressure sensitivity profile of the H_0.5_L_2_ resonator (e); Measured response and recovery times under 1.70 kPa stimulus (f); Sensitivity retention after 100 mechanical bending cycles (g); Stability following 12 h exposures at ‐20 °C and 40 °C (h). (i and j) Performance characterization of humidity‐sensing resonator unit: Resonance frequency shifts and *S*_11_ variations under varying relative humidity levels (i); Dynamic response behavior under controlled humidity variations (j). (k) Performance characterization of gas‐sensing resonator unit: Frequency shifts and *S*_11_ variations in response to NO_2_ concentrations from 0.2 to 16 ppm.

In practical applications, the relative position between the *T*_c_ and the resonator's *R*_c_ significantly influences *S*_11_, thereby affecting readout of multi‐stimuli signals. Electromagnetic simulations (Figure S23) reveal that the *S*_11_ of the resonator with the *R*_c_ positioned at the edge of the *T*_c_ is nearly twice that of the center‐aligned configuration (Figure 4a), owing to enhanced magnetic coupling from proximity effects between coil winding [50], highlighting the feasibility of using a single *T*_c_ for multimodal signals transmission in resonator array. To further evaluate *S*_11_ stability under positional variations, the *R*_c_ is laterally displaced by 20–30 mm from the *T*_c_ center (Figure 4b,c). Despite fluctuations in *S*_11_ amplitude, the resonance frequencies remain within narrow bands, demonstrating the large‐diameter *T*_c_’s resilience to misalignment.

To optimize the pressure sensing capability of the IWP wearable sensor, hydrogel‐based pressure‐sensing resonators with configurations H_0.5_ (without LYAH bottom layer), L_2_ (without HYH compression supporting interlayer and micro‐pyramidal patterned top layer), H_0.5_L_1_, H_0.5_L_2_, H_2_L_1_, and H_2_L_2_ were tested, where *x* in H*_x_* L*_x_* denotes layer thickness (mm). The H_0.5_‐based resonator exhibits a smaller saturated frequency shift (8.60 MHz) than the L_2_‐based resonator (22.70 MHz) but offers a wider detection range (0.72–16.80 kPa vs. 0.72–12.00 kPa) (Figure S24a,b,g and Table S4), indicating that the interlayer and top layer deliver robust structural support for high‐pressure endurance. While the softer and highly conductive bottom layer effectively fills the capacitor's dielectric region, amplifying capacitance changes and frequency shifts under low‐pressure stimuli (Figures S15 and S25). Based on the above analysis, the effect of each layer's thickness in the trilayer hydrogel on pressure‐sensing performance was systematically evaluated (Figure S24c–g). With the interlayer fixed at 0.5 mm, increasing the bottom layer thickness from 1 mm to 2 mm lowers the detection limit from 0.48 kPa (H_0.5_L_1_) to 0.24 kPa (H_0.5_L_2_), indicating that a thicker, softer LYAH bottom layer enhances sensitivity to low‐pressure stimuli. Conversely, increasing the interlayer thickness from 0.5 to 1 mm does not extend the detection range but reduces saturated sensitivity, as the stiffer HYH interlayer exhibits limited deformation under additional pressure [51]. Furthermore, as the overall thickness of the trilayer hydrogel increases, the saturated frequency shift consistently decreases (Table S4), due to increased capacitance and conductivity. Excessive capacitance causes an undesired downward shift in the initial resonance frequency, while elevated conductivity increases energy dissipation, reducing the *Q* factor and *S*_11_ amplitude, and ultimately compromising the resonator's sensitivity.

Collectively, the H_0.5_L_2_ trilayer hydrogel is selected as the optimal configuration. As pressure increases from 0.24 to 24.00 kPa, the H_0.5_L_2_ pressure resonator exhibits progressively enhanced frequency shifts, demonstrating its ability to detect a broad pressure range with high sensitivity (6.15 MHz·kPa^−1^ across 0.24–2.4 kPa, and 0.57 MHz·kPa^−1^ across 4.8–24.0 kPa) (Figures 4d,e, and S26). Notably, the experimental *S*_11_ spectra measured under various pressures further confirm that the pressure‐induced frequency response is dominated by the local capacitance change of the pressure‐sensitive resonator, while the resonant peaks of the humidity‐ and gas‐sensing units remain essentially unchanged (Figure S27 and Supporting Discussions 2.3). At 1.70 kPa, it demonstrates ultrafast response and recovery times of 5 and 6 ms (Figure 4f), enabled by rapid capacitance modulation in the LC circuit without reliance on internal charge transport mechanisms [52]. The resilient HYH interlayer also promoted fast structural recovery upon pressure release. Moreover, the resonance frequency shift (Δ*f*_P_) of the resonator remained stable after multiple bending cycles and repeated exposures to extreme temperatures (‐20°C – 40°C) or low/high pressures (0.24 and 15 kPa), highlighting its excellent mechanical durability and environmental stability (Figures 4g,h and S28a,b). The sensor maintained exceptional performance consistency even after 22 days of exposure to ambient room conditions (Figure S28c). Additionally, to further elucidate the pressure‐sensing mechanism, we experimentally characterized the relationship between applied pressure and the resulting changes in the electrical properties (specifically resistance and capacitance) of the trilayer hydrogel (H_0.5_L_2_) dielectric layer (Figure S29). Increasing pressure leads to an increase in capacitance and a decrease in resistance within the LC circuit, resulting in a corresponding reduction in both the resonance frequency (*f*_P_) and the *S*_11_.

To further assess the ambient moisture sensing capability of the IWP wearable sensor, the humidity‐sensing resonator unit incorporating zeolite‐based sensing layer was measured in a dynamic chamber, where resonance frequency shifts (Δ*f*_H_) were recorded across a relative humidity range of 2%–98% RH (Figures 4i and S31). The resonance frequency (*f*_H_) of humidity resonator exhibits a continuous upward shift with increasing humidity, due to water molecule adsorption on the zeolite layer, which enhances conductivity and reduces the effective capacitance of the resonator. Real‐time monitoring confirms a gradual and reversible frequency shift of the humidity resonator even at high humidity levels (98% RH) (Figure 4j). Sensitivity defined as the frequency change per 1% RH is linearly fitted across different humidity ranges, reaching 0.57 MHz·%RH^−1^ between 40% and 98% RH (Figure S31a). Notably, the humidity resonator does not saturate at 98% RH, highlighting the zeolite's strong water adsorption capacity. Besides, it exhibits periodic cyclic stability, response and recovery times of 614 s and 296 s, respectively, and maintains long‐term stability for 18 days (Figure S31b–d).

To extend the functionality of the IWP sensor beyond that of human skin, a gas‐sensing resonator unit incorporating metal oxide‐based sensing layer was designed. NO_2_ sensing tests reveal that the resonance frequency (*f*_G_) and frequency response shifts upward progressively with increasing gas concentrations (Figure 4k), aligning with simulation results. The low detection limit of 200 ppb enables continuous monitoring of the surrounding air quality and helps users avoid prolonged exposure to hazardous sub‐ppm levels of NO_2_. In addition, it is worth noting that the gas resonator fully recovers to its baseline after exposure to 16 ppm NO_2_ (Figure S32a). A linear fit of frequency shift (Δ*f*_G_) versus NO_2_ concentration shows a sensitivity of 1.74 MHz·ppm^−1^ across 0.2–2 ppm range (Figure S32b). Repeated exposures to 1 ppm NO_2_ produce consistent response curves, confirming excellent repeatability (Figure S32c). Selectivity test shows negligible responses to other gases, indicating strong anti‐interference performance (Figure S32d). The sensor maintains high sensitivity to 1 ppm NO_2_ for 15 days under ambient conditions, validating its long‐term stability (Figure S32e). Furthermore, to demonstrate the sensor's robustness in complex practical environments, target gas detection was performed in the presence of strong interfering backgrounds. The gas‐sensing resonator exhibited stable and consistent responses to 1 ppm NO_2_ even under the severe interference of 5 ppm NH_3_, 100 ppm CO, and high ambient humidity (80% RH) (Figures S33 and S34). This exceptional anti‐interference capability further ensures the reliability of the IWP sensor for olfactory monitoring. The exceptional NO_2_ sensing performance at room temperature is ascribed to the abundant oxygen vacancies (O_V_) on CdSnO_3_ surface (Figure S3d), which not only enhance gas adsorption but also facilitate efficient charge transfer [53]. This promotes the electron‐capture process of NO_2_ (NO_2_ + e^−^ →NO_2_^−^) (Figure 3h–j) [54], enabling the detection of trace‐level NO_2_ at room temperature with high selectivity. To evaluate reproducibility, three batches of IWP sensors were fabricated. They all showed highly consistent sensing performance and small response deviations, confirming the reliability and repeatability of our fabrication process (Figure S35).

### 2.5 Wearable Operation: Decoupled Perception of Superimposed Multi‐Stimuli

**FIGURE 5:**
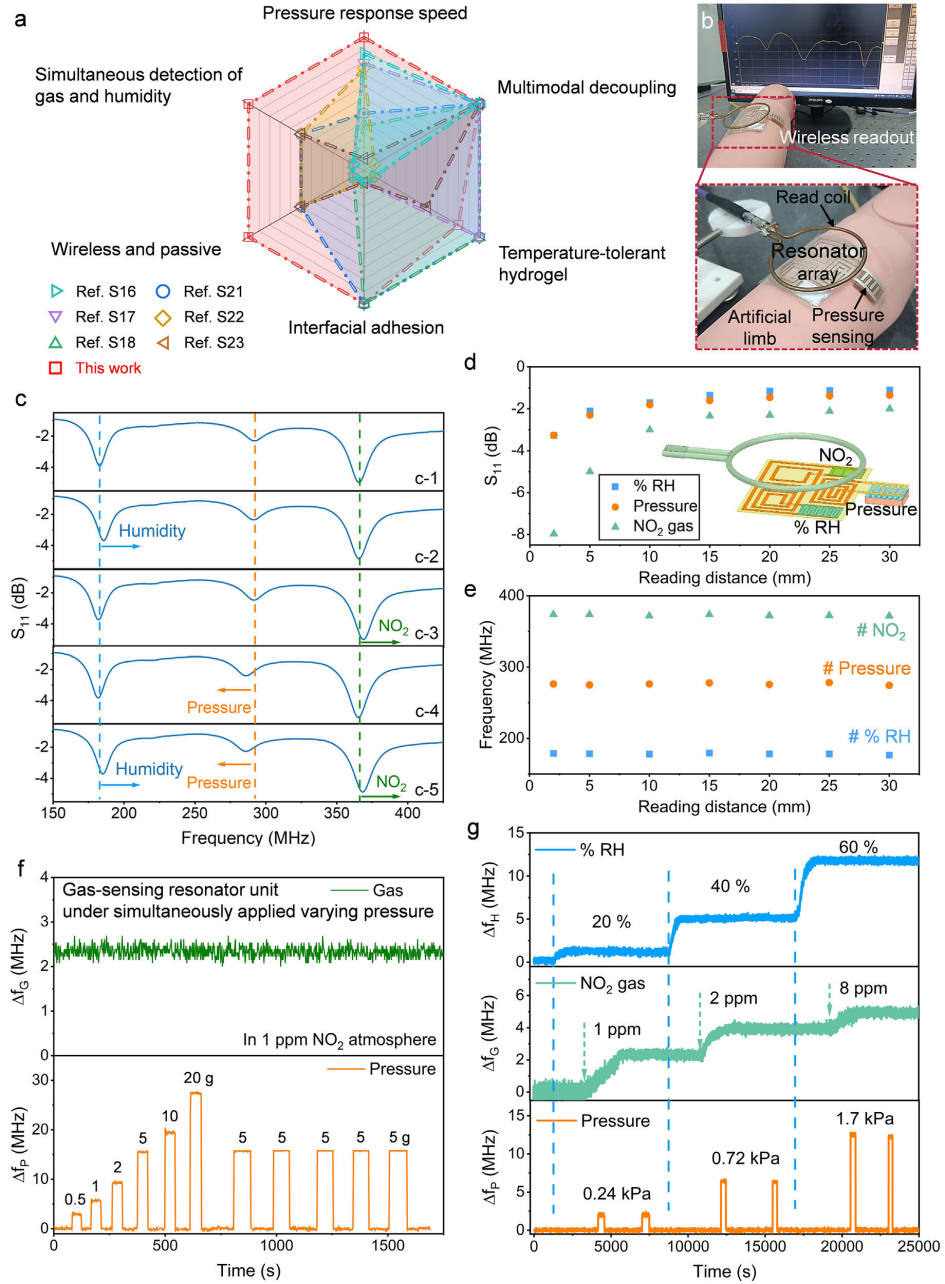
Multiplexed parallel readout capability of the IWP sensor. (a) Performance comparison among various multimodal wearable sensors. (b) Wearable demonstration showing the IWP sensor mounted on arm for real‐time monitoring (red dotted box: close‐up view). (c) RF spectral responses under individual and simultaneously applied stimuli of 30% RH humidity, 0.60 kPa pressure and 1.5 ppm NO_2_ stimulus. (d and e) Dependence of *S*_11_ parameters and resonance frequencies of the IWP sensor on the distance between the *T*_c_ and *R*_c_. (f) Simultaneous detection response for both NO_2_ (1 ppm) and applied pressure (0–24 kPa) stimulus. (g) Synchronous dynamic response curves of the humidity‐, pressure‐, and gas‐sensing units under simultaneously applied humidity levels (20%, 40%, and 60% RH), NO_2_ concentrations (1, 2, and 8 ppm), and cyclic gradient pressures (0.24, 0.72, and 1.7 kPa).

The IWP wearable sensor developed here outperforms conventional multimodal wearable sensors reported in previous literature (Figure 5a and Table S5), offering wireless passive operation, rapid response and recovery times, and effective signal decoupling for simultaneous detection of superimposed multi‐stimuli. The sensor patch can be conformally attached to the forearm for real‐time monitoring of skin pressure, as well as ambient humidity and NO_2_ levels (Figure 5b), with wireless communication reliably established over a few millimeters between the *T*_c_ and *R*_c_. For passive devices, the power required to achieve reliable RF coupling is as low as 3–5 mW, ensuring safe operation well below the specific absorption rate (SAR) limit of ∼1.6 mW g^−1^ and preventing heating issues on superficial skin tissues (Figure S36) [55]. Additionally, the sensor exhibits resonance frequencies in the 150–400 MHz range and operates exclusively via near‐field electromagnetic coupling with the *T*_c_.

Given the parallel readout architecture of the IWP sensor, cross‐coupling among resonator units was systematically evaluated. By spacing resonance frequencies over 93 MHz, effective decoupling is achieved with frequency shifts in one unit exerting negligible impact on others, finally suppressing mutual interference (the principles for determining the resonance frequency spacing are discussed in detail in Supplementary Discussion 2.2) [56]. To visually verify the simultaneous multiplexed readout capability, Figure 5c illustrates the RF spectral responses under individual and simultaneously applied humidity, pressure, and NO_2_ stimuli. Under individual stimulation, each stimulus induces a resonant‐frequency shift only in its corresponding resonator unit. When 30% RH humidity, 0.60 kPa pressure, and 1.5 ppm NO_2_ are simultaneously applied, the frequency shifts of the three sensing units remain confined to their respective resonators and are nearly identical to those obtained under single‐stimulus conditions, with almost no attenuation in the *S*_11_ amplitude (Figure S37). Furthermore, the signal loss during this simultaneous multiplexed readout process was quantitatively evaluated. The multiplexed readout induces only a negligible drop (less than 5%) in the *S*_11_ magnitude (Figure S38). This robust independent frequency tuning is similarly validated under dual‐stimulus combinations (Figure S39). Additionally, the wireless transmission stability of the IWP sensor was systematically evaluated under varying transmission distances between the external *T*_c_ and the sensor *R*_c_ (0–30 mm), bending angles (0°–90°), tilting angles (‐30°–30°), and skin‐temperature (32.2–38.4°C) (Figures 5d,e and S40–S45). Variations in transmission distance, bending, and tilting mainly change the mutual inductance and reflected impedance between *T*_c_ and *R*_c_, leading to expected fluctuations in the *S*_11_ amplitude (Supporting Discussions 2.4). However, the resonance frequency is primarily determined by the intrinsic inductance and capacitance of each LC resonator and is therefore largely independent of spatial coupling variations [57]. Consistently, the maximum frequency‐drift coefficients under bending and tilting are only 0.0078 MHz/° and 0.0226 MHz/°, respectively, corresponding to negligible equivalent stimulus offsets compared with the effective sensing ranges. In addition, within the skin‐temperature‐related range of 32.2–38.4°C, the temperature coefficients of the humidity‐, pressure‐, and gas‐sensing units are 0.00803, 0.1145, and 0.07746 MHz/°C, respectively, which correspond to equivalent perturbations of only 0.014% RH/°C, 0.019 kPa/°C, and 0.044 ppm/°C. Furthermore, even under variations in tilting angle (0° and 15°) and temperature (20°C and 40°C), the decoupled multimodal sensing performance remains robust and distinguishable (Figure S45). These results indicate that physical deformation and skin‐temperature fluctuations mainly affect the *S*_11_ amplitude rather than the resonance frequencies, thereby ensuring reliable wireless readout and decoupled multimodal sensing under practical wearable conditions.

To further validate the interference immunity among the resonator array, the IWP sensor was tested under varying pressures in the presence of 1 ppm NO_2_ (Figure 5f). The pressure‐induced frequency shift (Δ*f*_P_) of pressure‐sensing resonator increases with applied force, showing reproducible responses under a 5 g load, while the NO_2_ gas‐sensing resonance frequency (*f*_G_) remains stable, confirming mechanical load immunity. Additionally, ambient humidity as a potential interfering factor was systematically evaluated across different sensing modalities (Figures S46 and S47). Under varying humidity levels from 20% to 60% RH, the sensor reliably detects ambient humidity transitions (gradient Δ*f*_H_) as well as NO_2_ (1.5 ppm) and pressure (0.72 kPa) signals (consistent Δ*f*_G_ and Δ*f*_P_). This suggests that the responses of the IWP sensor to NO_2_ and pressure remain stable and repeatable under humidity fluctuations. Moreover, although the humidity‐sensing unit exhibits a relatively slow response, it remains suitable for long‐term wearable microclimate monitoring and does not substantially interfere with rapid pressure detection. To further evaluate the robustness of the IWP sensor under dynamically varying co‐existing stimuli, continuous real‐time measurements were conducted by simultaneously applying humidity levels of 20%, 40%, and 60% RH, NO_2_ concentrations of 1, 2, and 8 ppm, and cyclic gradient pressures of 0.24, 0.72, and 1.7 kPa (Figures 5g and S48). All three sensing units independently and accurately track their corresponding target stimuli. Notably, the fast‐response pressure‐sensing unit precisely captures transient mechanical loading signals without noticeable frequency drift induced by the simultaneously varying humidity and gas concentrations. Collectively, surpassing the capabilities of human skin and previously reported e‐skin devices, the IWP wearable sensor enables the first decoupled, simultaneous detection of pressure, humidity, and NO_2_ gas stimuli with high sensitivity and selectivity.

## 3 Conclusion

The integrated wireless passive wearable sensor patch introduced here is distinguished by its ability for rapid response (5 ms), stable performance across a wide temperature range (‐20°C–40°C), and capability to simultaneously detect multiple superimposed multi‐stimuli with signal decoupling. This sensor patch enables frequency‐division multiplexing of multiple resonators, serving as a key enabler for multimodal sensing applications. Meanwhile, wireless and real‐time operation, enabled by multiple resonators distributed on skin, facilitates omnidirectional and remote perception of multi‐stimuli. Comprehensive in situ experiments and simulations identify the operational mechanism of LC resonator array, supported by validated analytical models that guide the optimized design of compact and decoupled resonators, robust electromagnetic coupling system, and sensing layers. In comparison to conventional multimodal wearable sensors, the developed wearable sensor offers efficient multiplexed information transmission and enhanced perceptual capabilities with minimal mutual interference, enabling simultaneous detection of weak to broad‐range pressure (0.24–24 kPa), trace‐level NO_2_ (200 ppb), and dynamic humidity levels (2%–98% RH).

While this study demonstrates the development of a wireless, passive wearable sensor system with notable advantages in power efficiency and real‐time multi‐modal decoupled detection, the portability of the sensor system remains a key consideration. As noted, reliance on VNA for resonance spectrum detection may limit miniaturization and complicate field deployment. Recently, several commercially available handheld VNAs have emerged as a viable solution, offering exceptional portability with a typical frequency range of 1 MHz to 3 GHz, sufficient for most LC‐based sensor applications [58]. Utilizing the handheld VNA could enable practical system‐level portability, thereby enhancing the sensing system's adaptability for IoT‐based mobile platforms.

In summary, the proposed LC‐based multimodal sensor system is expected to significantly enhance the functionality and versatility of e‐skin, positioning LC resonator array as a promising platform for next‐generation wearable sensors.

## 4 Experimental Section

### 4.1 Preparing the Pressure‐Sensing Layer

#### 4.1.1 Synthesis of Hydrogel

Typically, the hydrogel precursor was prepared by sequentially dissolving a quantity of BA and CA, and 0.3 g of CS in PA solution, followed by addition of PVA (1 g), glycerol, and deionized water. After heating at 92°C for 3 h, the precursor underwent freeze‐thaw cycling (‐20°C, 20 h) for obtaining PVA‐CS hydrogel. Accordingly, a series of hydrogels with low Young's modulus (**BPG**, **BPCG**) and high Young's modulus (**CP**, **CG** and **CPG**) were prepared (See Supporting Note 1, and Table S1 for details).

#### 4.1.2 Fabricating the Trilayer Hydrogel

The gradient trilayer structure was fabricated through a sequential casting and lamination process. First, the **CPG** precursor was cast into a custom PTFE mold with micro‐pyramid array pattern (pyramid dimensions: 2 × 2 × 1.5 mm^3^; 0.5 mm spacing) and freeze‐thaw cycled to create HYH compression supporting interlayer and micro‐pyramidal patterned top layer. Subsequently, LYAH bottom layer was prepared using an identical process with **BPCG** precursor. The final pressure‐sensing trilayer structure was assembled by precisely laminating alternating HYH and LYAH layers with controlled thickness variations (See details in Supplementary Note 1).

### 4.2 Evaluations of Sensitive Materials

#### 4.2.1 Mechanical Experiments

Tensile tests were conducted using dumbbell‐shaped specimens (50 × 4 × 2 mm^3^) under uniaxial or cyclic loading at a constant strain rate of 50 mm min^−1^. The Young's modulus was extracted from the linear region (5%–20% strain) of the stress–strain curve, and toughness was calculated as the area under the curve. Compression tests were performed on cylindrical samples (Φ 12 × 10 mm^2^) at a rate of 10 mm min^−1^. All mechanical tests were repeated at least three times for consistency.

#### 4.2.2 Dielectric Property Characterization

The dielectric properties were evaluated using the coaxial probe method (Keysight Technologies). Powder samples, such as CdSnO_3_ and zeolite, were pressed into cylindrical pellets and placed at the bottom of a sealed test chamber. The probe was calibrated using the known dielectric constants of air and deionized water at 25 °C, and then vertically pressed against the sample surface to avoid air gaps (see Figure S22). NO_2_ concentrations were controlled using a custom dynamic gas mixing system, and dielectric spectra were recorded after 45 min of equilibrium at each target concentration. For hydrogels, dielectric measurements were performed directly without pelletization. Complete experimental protocols are detailed in Supporting Note 2 (Supporting Information).

### 4.3 Preparing the IWP Wearable Sensor

#### 4.3.1 Fabricating the LC Resonant Circuit Array

A 125 µm‐thick PET film was used as the substrate. LC circuit patterns were screen‐printed (200‐mesh stencil) on both sides using silver paste and thermally cured at 100 °C. For pressure‐sensing resonant unit, the front‐side planar spiral capacity antenna was electrically interconnected with back‐side interdigitated electrodes of capacitor through via holes filled with Ag paste, forming series‐connected LC resonant circuit.

#### 4.3.2 Integrating the Capacitive Sensing Layers on Resonator Array

After plasma‐treating the trimmed substrate, a 50 µL dispersion of gas‐ and humidity‐sensing materials (2 mg mL^−1^ in ethanol) was drop‐cast onto the interdigitated electrodes and dried at 100°C for 12 h, forming 20 µm thick capacitive sensing layers for the gas‐ and humidity‐sensing resonant units, respectively. Subsequently, the trilayer hydrogel was bonded to the backside interdigitated electrodes of the cantilever pressure‐sensing resonant unit, serving as capacitive element. Full IWP sensor fabrication protocols appear in the Supporting Note 1(1.5) (Supporting Information).

### 4.4 Simulation Analysis of Resonator Array

#### 4.4.1 Electromagnetic Simulation

The electromagnetic performance of the *T*_c_ and the IWP sensor was simulated in the frequency range of 100–500 MHz using the commercial software ANSYS HFSS. Based on the theory of resonant perturbation, variations in the dielectric properties of materials deposited on the electrodes induce shifts in the resonance frequency (Δ*f*_X_) and/or changes in the signal magnitude (Δ*S*_11_). The intensity of the perturbation depends on the dielectric characteristics of the material. By systematically adjusting the dielectric constant (*ε*) and the dielectric loss tangent (tan δ), the resulting trends in Δ*f*_X_ and Δ*S*_11_ were obtained. Detailed simulation steps and configurations are described in Supplementary Note 3 (Supporting Information).

#### 4.4.2 Finite Element Simulation

Finite element simulations of the pressure‐sensitive functional layers were conducted using COMSOL Multiphysics 6.2. A hyperelastic material model was established to represent the hydrogel‐based structure, utilizing identical geometric dimensions and material parameters as those of the actual functional layers. The simulation primarily focused on elucidating the relationship between the applied pressure and the resulting volumetric strain, stress distribution, as well as the displacement generated along the z‐axis. The simulation workflow involved the following key steps: geometry construction, material assignment, application of boundary conditions and external loads, meshing, and solution setup. Detailed procedures are provided in Supporting Note 3 (Supporting Information).

### 4.5 Multimodal Sensing Measurements

For wireless multi‐stimuli monitoring, the *T*_c_ was SMA‐connected to VNA (Keysight, P9375A) to interrogate the IWP sensor (Figure S30). Reflection‐mode measurements monitored *S*_11_ parameters throughout all experiments. The pressure characterization system comprised a motorized test platform (ESM303, Mark 10) integrated with a digital force gauge (M5‐5, Mark 10). Controlled pressure application was achieved using precision‐calibrated weights of varying magnitudes.

The gas and humidity sensing performances of the IWP sensor were evaluated using a custom‐built system consisting of VNA, *T*_c_ coil, sealed test chamber, and mass‐flow controllers (MFCs) (Figure S30). Detailed characterization methodologies for all performance metrics are available in Supporting Note 4 (Supporting Information).

### 4.6 Human Subject Statement

The photographs showing the wearable sensor attached to human skin were included solely for demonstration purposes. No physiological measurements, biological sample collection, or human subject research were conducted. Informed written consent was obtained from all participants for the publication of these images.

## Author Contributions

W.H., T.W., P.S., I.P., and G.L. conceived the concept and designed the overall research; W.H. and T.W. designed, developed, and characterized the sensing materials, sensor device and process; W.H., H.X., and T.W. investigated and performed the experiments; W.H., H.X., T.W., X.D., and L.Z. conducted the data analysis; W.H., T.W., P.S., S.C., I.P., and G.L. wrote the manuscript.

## Conflicts of Interest

The authors declare no conflicts of interest.

## Supporting information




**Supporting File**: adma74293‐sup‐0001‐SuppMat.docx.

## Data Availability

The data that support the findings of this study are available from the corresponding author upon reasonable request.
