# Supplementary material for: A Wireless, Passive Multimodal Wearable Sensor With Decoupled Sensing Capabilities
Source: Adv Mater. 2026 Jul 30;38(52):e74293. doi: 10.1002/adma.74293 (PMC13579182; doi:10.1002/adma.74293)
Supplement: Supplementary file 1 [file ADMA-38-e74293-s001.docx]

**Supporting Information**

**A Wireless, Passive Multimodal Wearable Sensor with Decoupled Sensing Capabilities**

Wenjiang Han^1^ | Heng Xiao^1^ | Tianshuang Wang^1,2*^ | Xiaoran Ding^1^ | Liupeng Zhao^1^ | Seokjoo Cho^3^ | Peng Sun^1,2*^ | Inkyu Park^3*^ | Geyu Lu^1,2*^

^1^ State Key Laboratory of Integrated Optoelectronics (JLU Region), College of Electronic Science and Engineering, Jilin University, 2699 Qianjin Street, Changchun 130012, China |

^2^ International Center of Future Science, Jilin University, 2699 Qianjin Street, Changchun 130012, China |

^3^ Department of Mechanical Engineering, Korea Advanced Institute of Science and Technology (KAIST), Daejeon 34141, Republic of Korea |

**Correspondence:**

T. Wang; E-mail: wangtianshuang@jlu.edu.cn; ID: orcid.org/0000-0003-3196-3005

P. Sun; E-mail: pengsun@jlu.edu.cn; ID: orcid.org/0000-0002-9509-9431

I. Park; E-mail: inkyu@kaist.ac.kr

G. Lu; E-mail: lugy@jlu.edu.cn

**Table of Contents**

1. Supplementary Methods ............................................................................................2

2. Supplementary Discussions......................................................................................12

3. Supplementary Tables .............................................................................................16

4. Supplementary Figures ............................................................................................23

5. Supplementary References.......................................................................................71

1. **Supplementary** **Methods**

**Supplementary** **Note 1. Preparing the wireless passive multimodal wearable sensor**

**1.1. Experimental material:** Poly (vinyl alcohol) (PVA, Mw 89,000-98,000, 99+% hydrolyzed, Sigma-Aldrich), Chitosan (CS, 200-800 cP, Sigma-Aldrich), Phytic acid (PA, 70% in water, Macklin), CdCl_2_ (Macklin), glycerin (Sinopharm), zeolite (Sinopharm); Boric acid (BA), Citric acid (CA), SnCl_4_·5H_2_O and NaOH were purchased from Aladdin. All reagents can be used without further purification.

**1.2. Preparation of CdSnO_3_ materials:** First, 10 mL of deionized water and 5 mL of ethanol were mixed and stirred. Then, SnCl_4_·5H_2_O (0.3506 g) and CdCl_2_ (0.1925 g) were sequentially added. The solution was magnetically stirred for 15 minutes. After that, 0.2104 g of citric acid monohydrate (C_6_H_8_O_7_·H_2_O), 0.4080 g of sodium hydroxide (NaOH), and 25 mL of deionized water were added, and stirring continued for 30 minutes. The resultant solution was then transferred into Teflon-lined autoclaves and crystallized at an oven of 180 °C for 12 hours. After the hydrothermal crystallization process, the solid product was isolated by centrifugation, thoroughly washed with distilled water and ethanol and then dried at 80 °C. Finally, the sample was calcined in air at 750 °C with a heating rate of 2 °C/min for 6 hours.

**1.3. Hydrogel synthesis**

*1.3.1. Preparation of the BPG organohydrogel:* First, 0.1 g of boric acid and 0.3 g of chitosan were added to 4 mL of phytic acid solution and stirred thoroughly. Then, 1.2 g of glycerol, 1 g of PVA, and 1 mL of deionized water were added. After then, the mixture was heated and stirred at 92 °C in a water bath for 3 h. The resulting organohydrogel precursor solution was poured into a mold, frozen at -20 °C for 20 h, and then thawed at room temperature to obtain the BPG organohydrogel.

*1.3.2. Preparation of the BPCG organohydrogel:* First, 0.1 g of boric acid, 0.05 g of citric acid, and 0.3 g of chitosan were added to 4 mL of phytic acid solution and stirred thoroughly. Then, 1.2 g of glycerol, 1 g of PVA, and 1 mL of deionized water were added. After then, the mixture was heated and stirred at 92 °C in a water bath for 3 h. The resulting organohydrogel precursor solution was poured into a mold, frozen at -20 °C for 20 h, and then thawed at room temperature to obtain the BPCG organohydrogel.

*1.3.3. Preparation of the CP hydrogel:* First, 0.2 g of citric acid and 0.3 g of chitosan were added to a mixed solution of 1 mL phytic acid and 3 mL deionized water and stirred thoroughly. Then, 1 g of PVA and 2 mL of deionized water were added. After then, the mixture was heated and stirred at 92 °C in a water bath for 3 h. The resulting hydrogel precursor solution was poured into a mold, frozen at -20 °C for 20 h, and then thawed at room temperature to obtain the CP hydrogel.

*1.3.4. Preparation of the CG organohydrogel*: First, 0.2 g of citric acid and 0.3 g of chitosan were added to a mixed solution of 1.2 g of glycerol and 3 mL of deionized water and stirred thoroughly. Then, 1.2 g of glycerol, 1 g of PVA, and 1 mL of deionized water were added. After then, the mixture was heated and stirred at 92 °C in a water bath for 3 h. The resulting organohydrogel precursor solution was poured into a mold, frozen at -20 °C for 20 h, and then thawed at room temperature to obtain the CG organohydrogel.

*1.3.5. Preparation of the CPG organohydrogel:* First, 0.2 g of citric acid and 0.3 g of chitosan were added to a mixed solution of 1 mL of phytic acid, 1.2 g of glycerol, and 2 mL of deionized water and stirred thoroughly. Then, 1.2 g of glycerol, 1 g of PVA, and 1 mL of deionized water were added. After then, the mixture was heated and stirred at 92 °C in a water bath for 3 h. The resulting organohydrogel precursor solution was poured into a mold, frozen at -20 °C for 20 h, and then thawed at room temperature to obtain the CPG organohydrogel.

**1.4. Fabrication of** **pressure-sensitive functional layers:** The CPG organohydrogel precursor, after water bath treatment, was cast into polytetrafluoroethylene pyramid patterned molds with interlayers of varying thicknesses. After the freeze–thaw process, HYH pressure-sensitive functional layers consisting of a micro-pyramid layer and interlayers of varying thicknesses were obtained. Each micro-pyramid in the pyramid layer has a base size of 2 × 2 mm^2^, a height of 1.5 mm, and a spacing of 0.5 mm. Similarly, the LYAH layer was obtained by pouring the BPCG organohydrogel, post water bath treatment, into molds of varying thicknesses, followed by a freezing-thawing process. Finally, LYAH layers of different thicknesses were bonded to HYH layers of different thicknesses to form hydrogel pressure-sensitive functional layers with different structures.

**1.5. Fabricating and integrating the resonator array:** First, an 8 × 8 cm PET film (thickness: 125 µm) was used as the substrate. The substrate was fixed onto a manual screen-printing platform using a 200-mesh stencil. Subsequently, 200 µL of silver paste was dispensed onto the stencil, and the antenna pattern was printed uniformly using a squeegee at a constant speed. After printing the front side of the device, the silver paste was cured in an oven at 100 °C. The back side was then fabricated following the same procedure. Vias were created at both ends of the electrodes and filled with conductive silver paste to electrically connect the top and bottom conductive layers, thereby forming a complete circuit that connects the planar spiral inductor in series with the planar interdigitated capacitance electrodes. The assembled device was further cured at 100 °C for 60 minutes. Finally, the excess substrate material was trimmed to complete the fabrication process. Following plasma treatment to render the surface of the planar interdigitated electrodes hydrophilic, 50 μL of an ethanol dispersion containing 2 mg mL^-1^ of humidity- and gas-sensitive materials was drop-cast onto the electrodes using a micropipette. The device was then placed in an oven and dried at 100 °C for 12 hours to form a humidity- and gas-sensitive film with a thickness of approximately 20 μm. Finally, a hydrogel-based pressure-sensitive functional layers was affixed onto the capacitive region of the pressure-sensing resonant unit, completing the fabrication of the IWP sensor.

**Supplementary** **Note 2. Other characterizations and testing of sensitive materials**

**2.1. Characterizations:** The microscopic morphology of the hydrogels was studied by scanning electron microscopy (SEM, Hitachi TM4000Plus and FESEM, JEOL JSM-7900F) at an acceleration voltage of 5 kV, in which the hydrogels were lyophilized for observation. The chemical components and structure over a range of 4000–400 cm^-1^ were measured by a Fourier-transform infrared spectroscopy spectrometer (FT-IR, Nicolet iS10). The crystalline information of all obtained samples was collected by X-ray powder diffraction (XRD, Rigaku D/Max-2550) with Cu-Kα radiation (λ = 1.5418 Å) in the 2θ range of 5-80°. X-ray photoelectron spectroscopy (XPS) studies were performed with a Mg Kα X-ray source (1253.6 eV). Differential scanning calorimetry (DSC, TA DSC25, USA) and thermogravimetric analysis/Derivative Thermogravimetry (TGA/DTG, Mettler TGA-3, Switzerland) of organic hydrogels from 25 to -80 °C and from 25 to 800 °C, respectively, were tested under nitrogen atmosphere at a rate of temperature change of 10 °C min^-1^. The dielectric constant test was operated on Keysight N1501SA-102. Testing the resistance and capacitance of the pressure resonator unit by the Keithley 7510.

**2.2.** **Mechanical experiments:** The tensile and compression tests of the hydrogels were conducted by an electric universal testing machine (AGS-X 500 N, Shimadzu, Japan) equipped with 500 N load cells at room temperature. The uniaxial and cyclic tensile tests were measured using dumbbell-shaped samples (50 × 4 × 2 mm^3^) at a speed of 50 mm∙min^-1^. The Young's modulus was obtained from the slope of the linear region (5-20% strain) from the stress–strain curves. The toughness was calculated from the area below the stress–strain curves. The hydrogel samples, with a diameter of 12 mm and a height of 10 mm, were used for compression tests with a compression speed of 10 mm min^-1^. Each test was repeated at least three times.

**2.3. Organohydrogel water retention measurement:** Organohydrogels were placed in airtight glass vials at a temperature of 25 °C and humidity of 33% RH (MgCl_2_ saturated salt solution) and their weights were recorded regularly. At least three sets of each sample were tested. The water retention property of the organohydrogels was defined as the weight ratio of the remaining mass to the initial mass.

**2.4. Conductivity measurement of hydrogels:** The electrical resistance of organohydrogel was measured by using a digital multimeter (DMM7510, Keithley). The organohydrogels were cut into a strip with a size of 15 mm (length) × 4 mm (width) × 2 mm (thickness). The conductivity (*σ*) of the organohydrogels was calculated by the following equation:

$\sigma=\frac{L}{Rs}$	(1)

where *L*, *R*, and *s* represented the length between adjacent electrodes, the resistance of the organohydrogels, and the cross-sectional area of the organohydrogels, respectively. Test at least three groups of each sample.

**2.5. Quantitative Evaluation of Hydrogel Adhesion:** The interfacial adhesion strength of the BPCG hydrogel was quantitatively evaluated via standard 180° peeling tests using a universal testing machine (AGS-X 500 N, Shimadzu, Japan) [S1]. Briefly, the hydrogel samples (width: *w* = 10 mm) were gently pressed onto various targeted substrates (glass, acrylic plates, and fresh porcine skin) under a 1 kg weight for 15 min to ensure stabilization. To prevent energy dissipation induced by the bulk elastic deformation of the hydrogel during the peeling process, a non-stretchable flexible polyethylene terephthalate (PET) film was firmly bonded to the back surface of the hydrogel. During the measurement, the PET-backed hydrogel was peeled off from the substrates at a constant peeling velocity of 50 mm/min. The peeling force was recorded as a function of displacement. The interfacial toughness (*T*) was quantitatively calculated using the following equation:

$T =\frac{2F}{w}$	(2)

where *F* is the average steady-state peeling force derived from the plateau region of the measured force-displacement curves, and *w* is the width of the hydrogel sample. At least three independent measurements were performed for each substrate.

**2.6. In situ dielectric probe measurement:** The dielectric properties were characterized using the coaxial probe method (Keysight Technologies). For solid-state samples (e.g., CdSnO_3_ powder), the material was first compressed into a smooth, flat-ended cylindrical pellet using a tablet press and gently placed at the bottom of the test chamber.

Use slim-profile connected to a VNA for testing, and calibrate using the known dielectric constants of air and deionized water at 25 °C. During measurement, the probe tip was aligned vertically and brought into firm contact with the pellet surface to eliminate any air gaps that could introduce error. The interface between the probe and chamber was sealed with an airtight O-ring to ensure measurement stability. The experimental setup is shown in Figure S22. The NO_2_ concentration inside the test chamber was precisely controlled using a dynamic gas mixing system (Same as the gas supply system in Supplementary Note 4 (1)). After stabilizing at each concentration point for 45 minutes, dielectric spectra were recorded using the Materials Measurement Suite software (N1500A). For organic hydrogel samples, the dielectric constant can be directly measured by the probe without the need for pressing.

**Supplementary** **Note 3. Simulating the IWP sensor**

**3.1. HFSS modeling and simulation**

*3.1.1.* Electromagnetic model for simulation: The electromagnetic response characteristics of the device were numerically simulated using the three-dimensional full-wave electromagnetic simulation software ANSYS HFSS. The simulation settings were configured as follows:

*3.1.2.* *Model construction and material definition:* The simulation model consisted of the IWP sensor and the transmission coil (*T*_c_). Detailed structural components, dimensions, and relative permittivity (*ε*) are listed in Table S6. The *ε* values for the hydrogel, zeolite, and metal oxide (CdSnO_3_) were experimentally obtained using a dielectric probe. The thickness of the silver electrode was determined by optical microscopy, while the electromagnetic parameters of other materials were sourced from the HFSS material database. The transmission coil *T*_c_ was modeled using a standard 6 mm^2^ copper wire, in accordance with national specifications. To accurately account for the parasitic effects of the SMA connector, 3 pF lumped capacitor was connected in parallel across the *T*_c_ input terminals.

*3.1.3.* *Boundary conditions and excitation configuration:* To emulate a free-space radiation environment, a cubic air box was constructed around the model, with its outer surfaces assigned as radiation boundaries. For calculation accuracy and efficiency, the side length of the air box is set to be two times the quarter of the wavelength (λ_0_) corresponding to the minimum frequency of 0.1 GHz plus the maximum size of the IWP sensor (100 mm) (Side length of air box = (2*λ_0_)/4+100 mm). 3 pF parasitic capacitor was modeled using a lumped RLC boundary, configured as a purely capacitive element (resistance = 0 Ω, inductance = 0 H, capacitance = 3 pF). Coil excitation was implemented via a lumped port with an impedance of 50 Ω, and the integration line was aligned radially along the coil.

*3.1.4.* *Solver settings and frequency sweep:* Simulations were performed at a solution frequency of 0.3 GHz using an adaptive meshing strategy. The convergence criteria were set to a maximum of 12 adaptive passes or a change in *S*-parameters of less than 0.02 between two consecutive iterations. The frequency sweep was carried out over the range of 0.1–0.5 GHz using a fast frequency sweep scheme, with a step size of 0.0002 GHz (200 kHz) to ensure accurate capture of resonance features.

*3.1.5.* *Data extraction and analysis:* Key simulation outputs included the *S*_11_ parameter (with emphasis on resonance frequency and magnitude) and the calculated quality factor (*Q*-factor). Additionally, the three-dimensional distributions of the electric (E-field) and magnetic (H-field) fields were analyzed to evaluate the electromagnetic field behavior of the IWP sensor. All simulations were completed on a workstation with 32 GB RAM.

**3.2. Calculation of the** ***Q*-factor:** The resonance point *S*_11_ magnitude is first converted to a voltage reflection coefficient (*Γ*).

$S_{11}(\mathrm{dB})=20\lg|\Gamma|$ (3)

By conservation of energy, the incident power (*P*_inc_) is the sum of the power transmitted to the load (*P*_trans_) and the reflected power (*P*_ref_) [S2].

$P_{inc}=P_{trans}{+ P}_{ref}$	(4)

$P_{trans}{/P}_{inc}=1-|{\Gamma|}^{2}$	(5)

At the half-power point, the power transmitted to the load drops to half of the resonant point (P_trans-max_/2). Thus, the reflection coefficient (*Γ*_th_) and *S*_11th_ at the half power point are:

$\Gamma_{th}=\sqrt{\left( 1-P_{trans-max}{/2P}_{inc} \right)}$	(6)

$S_{11th}(\mathrm{dB})=20\lg|\Gamma_{th}|$	(7)

The upper and lower cutoff frequencies (*f*_2_, *f*_1_) are measured from *S*_11th_ on the *S*_11_ curve.

${\Delta f}_{FWHM}=f_{2}-f_{1}$	(8)

${Q\approx\frac{f_{X}}{\Delta f}}_{\mathrm{FWHM}}$	(9)

**3.3. Finite element simulation**: The solid mechanics module was employed to characterize the mechanical behavior of the hydrogel using a two-parameter Mooney–Rivlin hyperelastic model. The material parameters, *C*_10_ and *C*_01_, were extracted by fitting the experimental stress–strain data of the hydrogel under uniaxial tension to the Mooney–Rivlin constitutive equation using the least-squares method. The boundary conditions were set as follows: a fixed constraint was applied to the bottom surface of the pressure-sensitive functional layer to simulate its adhered state, while a vertically downward load was applied to the top surface by the substrate to represent the actual working load conditions. To enhance the computational accuracy in critical regions, a local mesh refinement strategy was implemented around the tips of the micro-pyramids. All material parameters used in the finite element analysis are summarized in Table S7.

**Supplementary Note 4: IWP sensor for wireless** **multi-stimuli monitoring**

**4.1. Evaluation of gas and humidity sensing performances:** The gas and humidity sensing performances of the IWP sensor were evaluated using a custom-built system consisting of VNA, *T*_c_ coil, sealed test chamber, and mass-flow controllers (MFCs) (Schematic diagram of the sensor measurement system is shown in Figure S30). In detail, the IWP sensor was affixed to the inner surface of the glass lid of a 500 mL cylindrical glass test chamber; Then, a silicone sealing ring was placed between the glass lid and the chamber, and the assembly was clamped tightly to ensure an airtight seal. To evaluate gas-sensing performance, various gases—such as NO_2_—were introduced into the chamber. All target gases were commercially supplied as pre-diluted mixtures in dry air (nitrogen–oxygen mixture with 99.99% purity, V_nitrogen_ : V_oxygen_ = 8 : 2) at concentrations of 100 ppm. The final concentration of each analyte gas (typically 10 ppm) was precisely controlled by adjusting the flow rates of the analyte and carrier gases (dry or humid air) using MFCs. Additionally, humidity sensing tests were conducted by generating a controlled humid environment. Dry air was passed through a glass bottle containing wet pink diatomite (6201, 40-60 mesh) to produce humidified air (equation 10), which was subsequently mixed with diluted NO_2_ gas. The humidity was monitored using a calibrated hygrometer installed inside the sensing chamber.

Prior to testing, the sensor was preconditioned by flowing dry synthetic air over it for 24 hours to stabilize its baseline and minimize the influence of ambient environmental factors on the sensing material. During testing, the entire chamber was placed on a 10 cm-thick microwave-absorbing foam to suppress external electromagnetic interference. The *S*_11_ resonance frequency shift was monitored every 1 second using a custom LabVIEW program.

$\mathrm{RH}=\frac{V_{Humid air}}{V_{Dry air}+V_{Humid air}+V_{Target gas}}\times100\%$	(10)

Typically, the relative humidity of the entire inflow air was controlled by adjusting the ratio of dry air supplied through MFC-1 and wet air through MFC-2. The total flow rate was maintained at 1000 sccm. For example, in order to achieve 20% RH inside the chamber, adjust the flow rate of MFC-1 to 800 sccm and MFC-2 to 200 sccm. The target gas concentration in the chamber is controlled by injecting target gas (e.g., NO_2_) with 0% RH into MFC-3. For example, when RH is to be set to 20% and NO_2_ concentration to 1 ppm, MFC-1 = 790 sccm, MFC-2 = 200 sccm, and MFC-3 = 10 sccm are set. At this time, the total flow rate of dry air is MFC-1 + MFC-3 = 800 sccm, and the flow rate of wet air is 200 sccm, so the RH on the overall basis is maintained at approximately 20%, and the target gas concentration is maintained at approximately 1 ppm.

**4.2. Wireless transmission stability tests:** The wireless transmission stability of the IWP sensor was systematically evaluated under different readout distances, bending angles, tilting angles, and temperatures. The readout distance was defined as the vertical distance between *T*_c_ and the *R*_c_. For the bending test, the IWP sensor was bent to different angles of 0°, 15°, 30°, 45°, 60°, and 90° using a clamp. For the tilting test, the IWP sensor was mounted on a 1-mm-thick flat glass substrate and fixed onto a rotation stage to eliminate bending-induced effects. The tilting angle between the sensor plane and the external readout coil was adjusted from -30° to 30°, while the readout distance was maintained at 18 mm. For temperature evaluation, the IWP sensor was positioned above an inverted Petri dish on a heating stage. The sensor temperature was regulated by the heating stage and measured using an infrared thermometer. For all tests, the corresponding *S*_11_ spectra were acquired using the same RF readout system.

**4.3. Response and recovery times:** The response and recovery times were defined as the time required for the resonance frequency to reach 90% of the total frequency shift (Δ*f*_X_, including Δ*f*_H_, Δ*f*_P_, or Δ*f*_G_) when transitioning from the initial frequency without stimulus (*f*₀) to the stabilized frequency under stimulus (*f*_X_, including: *f*_H_, *f*_P_, or *f*_G_), and vice versa.

${\Delta f}_{X} =|f_{X}-f_{0}|$	(11)

**4.4. Definition of sensor sensitivity:** The frequency shift (Δ*f*_X_) of the sensor in response to variations in pressure, gas concentration, and humidity is fitted to a linear calibration curve. Sensitivity (*S*) is defined as the frequency shift per unit concentration change Δ*C* (Δ*H*, %RH; Δ*P*, kPa and Δ*G*, ppm) in the external stimulus (pressure, humidity, and gas), with units of MHz·%RH⁻¹, MHz·kPa⁻¹ and MHz·ppm⁻¹, respectively.

$S=\frac{{\Delta f}_{X}}{\Delta C}$	(12)

2. **Supplementary** **Discussions**

**Supplementary Discussions 2.1. Detailed composition and analytical comparison of hydrogels**

Specifically, introducing boric acid and phytic acid yielded a low Young’s modulus adhesive hydrogel, while incorporating citric acid and phytic acid produced a high Young’s modulus hydrogel (Figure 2a, and Figure S5 and Table S1, 2). This is attributed to the fact that citric acid, with its higher number of hydrogen bond donors, imparts a greater cross-linking density. In contrast, although boric acid can form a three-dimensional hydrogen-bonding network, its lower hydrogen bonding strength and density make it more suitable for applications requiring reversible cross-linking. Interestingly, although the phosphate group in phytic acid promotes strong electrostatic and hydrogen bonding interactions that enable adhesion [S3], only the **BPG** organohydrogel exhibits robust adhesion, whereas the **CPG** organohydrogel lacks adhesion. This is because citric acid predominantly serves as the cross-linker in **CPG** organohydrogel, the excessive cross-linking density could restrict molecular chain mobility and limits the availability of interfacial reactive sites, resulting in diminished adhesion. Based on above analysis, to enable highly sensitive detection of surface pressure while preventing mechanical damage during movement, the organohydrogel with low Young’s modulus and robust adhesion was developed by incorporating a little amount of citric acid into the **BPG** organohydrogel synthesis system, resulting in the preparation of **BPCG** organohydrogel. The **BPCG** organohydrogel demonstrates robust adhesion, exceptional toughness, and outstanding stretchability, ensuring strong, conformal adhesion to knuckle skin—whether straightened or bent—as well as to glass and acrylic substrates (**Fig. 2**b-1–b-4). Furthermore, the **BPCG** organohydrogel not only exhibits strong adhesion, but also withstands large tensile deformation and can support a 500 g tensile load (**Fig. 2**b-5–b-7).

**Supplementary Discussions 2.2**

To determine the frequency spacing, we consider three constraints. First, due to the finite dimensions of the receiver coils, excessively small resonant-frequency spacing can induce signal perturbation between adjacent resonators. According to classical resonator design principles, the spacing between adjacent resonant peaks should exceed three times the resonance bandwidth to minimize electromagnetic crosstalk [S4, S5]. Therefore, the initial resonance frequencies were carefully separated by optimizing the inductor and capacitor geometries of each sensing unit. Second, the frequency spacing must accommodate the maximum spectral shifts under practical operating conditions. In our system, the maximum response-induced shifts for humidity, pressure, and gas sensing are about 32, 30, and 5 MHz, respectively. Finally, on this basis, an additional frequency interval should be reserved to accommodate possible baseline drift. The 93 MHz frequency spacing was determined based on the resonance bandwidth, the maximum sensing-induced frequency shifts, and the possible baseline drift of the sensing array under practical operating conditions.

**Supplementary Discussions 2.3**

In the idealized electromagnetic simulation, slight adjustments of the pressure-sensitive port (*d*_2_) can perturb the global electromagnetic field distribution, leading to small variations in parasitic capacitance and mutual inductance among the densely packed resonators. These effects are manifested as minor shifts in adjacent resonant peaks in the simulation, which is consistent with classical electromagnetic coupling theory [S2]. However, in practical measurements, several physical factors significantly suppress such theoretical crosstalk (Figure S27). First, the parasitic coupling between spatially separated coils decays rapidly with distance and is further weakened by the non-ideal electromagnetic environment. Second, the PET substrate introduces intrinsic dielectric loss with a finite loss tangent, which dissipates stray fields and attenuates fringe-field propagation, thereby effectively reducing inter-resonator coupling [S6].

As a result, the weak coupling predicted by simulations becomes negligible in real experimental measurements. The observed frequency response is therefore dominated by the local capacitance change of the pressure-sensitive resonator, while the resonant peaks of the humidity- and gas-sensing units remain essentially unchanged. Thus, the designed frequency-division spacing between resonators minimizes spectral overlap and improves selectivity, achieving reliable decoupled sensing under practical operation conditions

**Supplementary Discussions 2.4. Theoretical Analysis of Electromagnetic Coupling and Wireless Transmission Stability**

The wireless transmission stability of the IWP sensor was evaluated under varying transmission distance between the *T*_c_ and *R*_c_ (0–30 mm), bending angles (0°–90°), and tilting angles (-30°–30°) (Figure 5d,e and Figure S40-43). The results show that variations in transmission distance, bending angle, and tilting angle alter the mutual inductance between the transmitter coil and receiver coil, leading to expected fluctuations in the *S*_11_ amplitude. However, the resonance frequency is theoretically determined by the intrinsic inductance and capacitance of the LC resonator and is therefore largely independent of spatial coupling variations [S7]. As a result, the measured resonance frequencies remain nearly unchanged, ensuring reliable wireless readout under complex physical deformations.

The *S*_11_ reflection coefficient is fundamentally determined by the impedance matching between the input impedance observed at the readout coil *Z*_in_ and the characteristic impedance of the system (*Z*_0_, typically 50 Ω) [S8]. As *Z*_in_ approaches *Z*_0_, the impedance matching improves, leading to minimal energy reflection and a deeper *S*_11_ dip (i.e., a larger amplitude):

$S_{11}\left( \mathrm{dB} \right)=20lg\left| \frac{Z_{in}-Z_{0}}{Z_{in}+Z_{0}} \right|$	(13)

According to equivalent circuit theory, the total input impedance (*Z*_in_) observed by the readout coil is the sum of its self-impedance (*Z*_1_) and the reflected impedance (*Z*_r_) coupled from the receiver coil [S9]. At the resonance point:

$Z_{r}=\frac{{(\omega M)}^{2}}{R_{2}}$	(14)

$Z_{in}=Z_{1}+Z_{r}=Z_{1}+\frac{{(\omega M)}^{2}}{R_{2}}$	(15)

where *ω* is the angular frequency, and *M* is the mutual inductance between the two coils. The mutual inductance can be expressed as [S7]:

$M=K\sqrt{L_{1}L_{2}}$	(16)

Here, *L*_1_ and *L*_2_ represent the self-inductances of the transmission coil and the receiver coil, respectively, which are determined by the number of turns and geometric dimensions of the coils. Consequently, the mutual inductance *M* depends on the spatial coupling coefficient *k* between the coils. When the sensor undergoes tilting (at an angle of *θ*) or bending, the spatial coupling weakens, and the effective magnetic flux area penetrating the receiver coil decreases following a cosine relationship (which simplifies under the ideal parallel coil model as follows):

$M\propto cos\theta$	(17)

Although dynamic changes in spatial posture (e.g., tilting, bending, or misalignment) lead to a significant decrease in the mutual inductance *M* and a subsequent attenuation of the *S*_11_ amplitude. But the resonance frequency of the sensor:

$f_{0}=\frac{1}{2\pi\sqrt{L_{X}C_{X}}}$	(18)

*f*_0_ is determined by the inherent parameters of the sensor itself and is independent of the mutual inductance *M* brought by the transmission coil or the coupling coefficient *k*. Consequently, even if bending and tilting of the sensor cause prominent fluctuations in the *S*_11_ signal amplitude, the resonance frequency will not experience noticeable baseline drift.

**3. Supplementary Tables**

**Table S1.** Compositions of various hydrogels.

| **Samples** | **PVA (g)** | **CS (g)** | **CA (g)** | **BA (g)** | **PA (mL)** | **Glycerin (g)** | **DI (mL)** |
| --- | --- | --- | --- | --- | --- | --- | --- |
| **BPG** | 1 | 0.3 | 0 | 0.1 | 4 | 1.2 | 1 |
| **BPCG** | 1 | 0.3 | 0.05 | 0.1 | 4 | 1.2 | 1 |
| **CP** | 1 | 0.3 | 0.2 | 0 | 1 | 0 | 5 |
| **CG** | 1 | 0.3 | 0.2 | 0 | 0 | 2.4 | 4 |
| **CPG** | 1 | 0.3 | 0.2 | 0 | 1 | 2.4 | 3 |

**Table S2.** Hydrogels formulations and their mechanical profiles.

| **Hydrogel** | **Crosslinkers** | **Glycerol** | **Tensile/compressive Modulus (kPa)** | **Toughness (MJ·m^-3^)** | **Adhesion** |
| --- | --- | --- | --- | --- | --- |
| **BPG** | Boric acid + Phytic acid | ✓ | 12.5/15.3 | 1.6 | High |
| **BPCG** | Boric acid + Phytic acid + Citric acid | ✓ | 17.6/34.7 | 2.2 | High |
| **CP** | Citric acid + Phytic acid | ✗ | 40.6/116.7 | 1.7 | Poor |
| **CG** | Citric acid | ✓ | 59.4/220 | 3.1 | Low |
| **CPG** | Citric acid + Phytic acid | ✓ | 1233.6/313.3 | 8.3 | Low |

**Table S3.** *Q* factors of the IWP sensor at varying communication distances (*d*_1_) between the *T*_c_ and *R*_c_.

| ***d*_1_ (mm)** | ***Q* factor of *f*_H_** | ***Q* factor of *f*_P_** | ***Q* factor of *f*_G_** |
| --- | --- | --- | --- |
| **8** | 66.89 | 171.34 | 133.10 |
| **14** | 43.67 | 116.23 | 79.516 |
| **20** | 34.58 | 114.67 | 60.65 |
| **26** | 53.54 | 180.75 | 126.46 |

**Table S4.** Pressure sensing performance and parameters of different pressure-sensitive functional layers of IWP sensors.

| **Sample** | **Detection range**  **(kPa)** | **Saturation response value (MHz)** | **Integral device thickness (mm)** |
| --- | --- | --- | --- |
| **H_0.5_** | 0.72-16.80 | 8.6 | 2 |
| **L_2_** | 0.72-12.00 | 22.7 | 2 |
| **H_0.5_L_1_** | 0.48-16.80 | 37.9 | 3 |
| **H_0.5_L_2_** | 0.24-24.00 | 30.3 | 4 |
| **H_2_L_1_** | 0.48-16.80 | 27.3 | 4.5 |
| **H_2_L_2_** | 0.48-24.00 | 26.9 | 5.5 |

**Table S5.** Comparison of reported multimodal sensing systems.

| **Pressure response time (ms)** | **Interfacial adhesion** | **Multimodal decoupling** | **Wireless and passive** | **Temperature**  **-tolerant hydrogel** | **Simultaneous detection of gas and humidity** | **Refs.** |
| --- | --- | --- | --- | --- | --- | --- |
| --- | --- | Yes | Wireless / passive | --- | --- | [S9] |
| 5.6 | --- | Yes | Wireless | --- | --- | [S10] |
| 44 | --- | Yes | --- | --- | --- | [S11] |
| 70 | --- | Yes | Passive | --- | --- | [S12] |
| 220 | Yes | Yes | --- | -20 ~ 60 °C | --- | [S13] |
| --- | --- | Yes | --- | --- | --- | [S14] |
| 1000 | Yes | Yes | --- | RT ~ 52 °C | Humidity | [S15] |
| 190 | Yes | Yes | Wireless | RT ~ 50 °C | Humidity | [S16] |
| 80 | --- | --- | --- | --- | Humidity | [S17] |
| --- | --- | Yes | Wireless | --- | Humidity | [S18] |
| --- | --- | Yes | Wireless | --- | Humidity | [S19] |
| 97 | --- | --- | Passive | --- | --- | [S20] |
| 90 | --- | --- | --- | RT ~ 70 °C | Humidity | [S21] |
| --- | --- | Yes | Wireless /passive | --- | Humidity | [S22] |
| --- | --- | Yes | Wireless /passive | --- | Humidity | [S23] |
| --- | --- | --- | Wireless / passive | --- | Humidity | [S24] |
| 5 | Yes | Yes | Yes | -20 ~ 50 °C | Gas and humidity | **This work** |

**Table S6.** HFSS simulation parameters for IWP sensor.

| **Structural components** | **Material** | ***ε*** | **Size (mm)** |
| --- | --- | --- | --- |
| **PET flexible**  **substrate** | Polyethylene terephthalate | 4.3 | Thicknesses: 0.125 |
| **Electrode** | Silver | - | Thicknesses: 0.02 |
| **Gas-sensitive functional layer** | CdSnO_3_ | 4.0 | 10×7×0.1 |
| **Pressure-sensitive functional layer** | Organohydrogel | 8.0 | 17×12×2  17×12×0.5 |
| **Humidity-sensitive functional layer** | Zeolite | 2.4 | 18.5×12×0.1 |
| ***T*_c_** | Copper | - | Calibre: 60;  2.76 (thick) |
| **Input port parasitic capacitance** | Polyamide | 4.3 | 9×6.5×2.88 |
| **Test chamber lid** | Glass | 5.5 | 100×100×1 |
| **Air box** | Vacuum | 1.0 | 1600×1600×1600 |

All dimensions of the substrate and electrodes are shown in Figure S19.

**Table S7.** COMSOL simulation parameters for pressure-sensing resonator.

| **Structure** | **Parameters** | **Units** | **Value** |
| --- | --- | --- | --- |
| LYAH | Density | g/cm^3^ | 1.2 |
|  | Compressive modulus | kPa | 34.0 |
|  | Poisson ratio |  | 0.46 |
|  | *C*_10_ | kPa | 10 |
|  | *C*_01_ | kPa | -1 |
|  | bulk modulus | MPa | 0.1 |
| HYH | Density | g/cm^3^ | 1.5 |
|  | Compressive modulus | kPa | 313.3 |
|  | Poisson ratio |  | 0.46 |
|  | *C*_10_ | MPa | 1.5 |
|  | *C*_01_ | MPa | 0.4 |
|  | bulk modulus | MPa | 2 |

**4. Supplementary Figures**

**Figure S1.** **SEM images**. (a, b) clinoptilolite zeolite, (c, d) CdSnO_3_, (e) BPG, (f) BPCG, (g) CP, (h) CG, and (i) CPG hydrogels.

**Figure S2. XRD patterns.** (a) clinoptilolite-type zeolite, (b) CdSnO_3_.

**Figure S3.** **XPS spectra.** (a) full spectra of zeolite and CdSnO_3_, (b) Al 2p and (c) Si 2p for zeolite, as well as (d) O 1s, (e) Sn 3d and (f) Cd 3d for CdSnO_3_.

**Figure S4.** **FT-IR spectra of** **BPG, BPCG, CP, CG, and CPG hydrogels.** There is a broad peak at 3270 cm^-1^ ascribed to the overlapping symmetric stretching vibrations of PVA hydroxyl and CS amino groups [S25], and a signal at 1647 cm^-1^ ascribed to the amide I band of CS [S26].

**Figure S5. BPG, BPCG, CP, CG, and CPG hydrogels in the precursor solution transform into stable gels following water bath-induced polymerization.** For comparison, we prepared **CP** hydrogel without glycerol and observed rapid dehydration under dry conditions, leading to the complete loss of both mechanical integrity and pressure sensing capability. It can be found that the **CP** hydrogel rapidly loses moisture, dries out, and becomes fixed within the mold.

**Figure S6.** **Adhesion performance of the hydrogel.** (a) Adhesion strength versus displacement curves of the hydrogel peeled from various representative substrates (acrylic, glass, and pigskin) measured via standard 180° peeling tests. (b) The calculated interfacial toughness of the hydrogel on the corresponding substrates.

**Figure S7. The water retention tests of BPCG** **and** **CPG organohydrogels.** Photographs of organohydrogels (a) before and (b) after being exposed to 50 °C heat condition for 12 h, and (c) corresponding residual mass ratios. Scale bar: 20 mm. To evaluate the dehydration resistance of the organohydrogels, we exposed **BPCG** and **CPG** organohydrogels to a hot and dry condition (50 °C for 12 hours). After exposure, they retained 88.1% and 77.6% of their original mass, respectively, while maintaining their initial sizes.

**Figure S8.** **Photographs of BPCG and CPG organohydrogels under various bending angles (30°, 90°, 180°) after extreme temperature exposure**: (a, b) freezing at -20 °C for 24 h and (c, d) heating at 50 °C for 24 h. After 24 h at 50 °C/-20 °C, **BPCG** and **CPG** organohydrogels demonstrated excellent bending resilience (up to 180°) due to glycerol’s ice-crystallization suppression effect through water hydrogen bond disruption [S27].

**Figure S9. TGA and DTG curves at the temperature range of 25–800 °C.** (a) **BPCG** and (b) **CPG** organohydrogels. The thermogravimetric analysis (TGA) and derivative thermogravimetry (DTG) analyses demonstrated that **BPCG** and **CPG** organohydrogels could retain free water more effectively below 60 °C, showing slower evaporation rates.

**Figure S10. Differential scanning calorimetry (DSC) curves of BPCG and CPG organohydrogels at the temperature range of -80–25 ^o^C.** According to the differential scanning calorimeter (DSC) analysis, at cryogenic temperatures (-63 ^o^C), the absence of exothermic peaks—coupled with only a glass transition—confirmed suppressed ice formation due to reduced free-water content, further validating their freeze-resistant properties [S28].

**Figure S11.** **Residual mass ratio of BPCG and CPG organohydrogels during ambient storage (20 °C and 35% RH) over time.** Long-term water retention testing conducted under ambient conditions (20 °C and 35% RH) over a period of 20 days revealed high weight retention for **BPCG** (94.3 wt%) and **CPG** (91.2 wt%) organohydrogels.

**Figure S12.** **Schematic illustrations and corresponding optical photographs of the optimal trilayer hydrogel (H_0.5_L_2_) pressure-sensing layer.** The cross-sectional view (left) and top view (right) quantitatively detail the architectural dimensions, including the low Young's modulus adhesive hydrogel (LYAH) bottom layer (2.0 mm thick), the high Young's modulus hydrogel (HYH) interlayer (0.5 mm thick), and the 5 × 8 truncated micro-pyramidal array (base: 2.0 × 2.0 mm², top: 0.7 × 0.7 mm², height: 1.5 mm, and inter-pyramid spacing: 0.5 mm).

**Figure S13. Thickness statistics of the molded H_0.5_L_2_ pressure-sensing layer.** Statistical thickness measurements of the individual structural components: the micro-pyramids top layer, the HYH interlayer, and the LYAH bottom layer. Error bars represent the standard deviations of multiple measurements.

**Figure S14.** Simulated distributions of strain in pressure-sensitive functional layers at different pressures using COMSOL.

**Figure S15.** **Finite element simulation modeled the relationship curves with applied pressure for the pressure-sensitive functional layer.** (a) displacement and (b) volume strain.

**Figure S16.** Finite element simulations of stress distribution in pressure-sensitive functional layer at low and high pressures.

**Figure S17. (**a) Correlation between the contact area between the micro-pyramid tip and PET substrate and the applied pressure. (b) Correlation between the volume change of air within the micro-pyramid gap and the applied pressure.

**Figure S18.** The equivalent circuit of LC resonator array.

**Figure S19.** The dimension of IWP sensor (units: mm).

**Figure S20.** **RF spectra of the LC resonators measured before and after coating with corresponding sensing layers.** (a) humidity, (b) NO_2_, and (c) pressure.

**Figure S21.** Simulated and measured RF spectra of the IWP sensor before and after deposition of pressure-, humidity-, and gas-sensing layers.

**Figure S22. *in situ* dielectric probe test system evaluates the material’s dielectric constant under varying gas concentrations (or humidity levels).** The material, pressed into sheet, is characterized using a custom-built system consisting of sealed test chamber, and mass-flow controllers (MFCs).

**Figure S23. Simulated magnetic field distributions of the LC resonator via HFSS.** (a) inductance coil located directly beneath the center of the reader coil; (b) inductance coil positioned beneath the edge of the reader coil. Scale bar: 30 mm.

**Figure S24.** Sensitivity versus pressure curves of pressure-sensing resonant sensors with different thicknesses of pressure-sensing layers.

**Figure S25.** Conductivity of BPCG and CPG organohydrogels.

**Figure S26.** Fitted curves of LC pressure-sensing resonator’s sensitivity across the pressure range of 0.24-24.0 kPa.

**Figure S27.** RF spectral responses of the IWP sensor under pressure levels from 0 to 7.2 kPa at 40% RH. The results confirm that pressure induces a resonance-frequency shift only in the pressure-sensing unit, while the other resonance peaks remain unaffected.

**Figure S28. Evaluation of the durability and long-term environmental stability of the pressure-sensing resonator unit.** (a, b) The resonance frequency shifts (Δ*f*_P_) at (a) a low pressure of 0.24 kPa and (b) a high pressure of 15 kPa during the 300-cycle loading-unloading test. (c) The long-term sensing stability testing over a period of 22 days.

**Figure S29.** (a) Photograph of the electrical properties measurement system; Corresponding plots showing the variation of (b) resistance and (c) capacitance in the capacitive region of the H_0.5_L_2_ pressure-sensing resonator under different applied pressures.

**Figure S30.** **Dynamic sensing characterization system. (**a) Schematic of the testing platform for simultaneous gas- and humidity-sensing evaluation; **(**b) Experimental setup showing the testing chamber, where the IWP sensor is wirelessly powered and interrogated through the VNA’s single-port connection to the reading coil. The system enables real-time adjustment of both target gas concentration and chamber humidity levels.

**Figure** **S31. Humidity-sensing performance of the IWP sensor.** (a) Fitted curve of IWP sensor sensitivity versus 40–98% RH. (b) Repeatability measurement and (c) dynamic response characteristic of the IWP sensor towards 50% RH. (d) Long-term stability of the IWP sensor was evaluated over 18 days under 20% RH to assess changes in sensitivity.

**Figure S32. NO_2_ gas-sensing performance of the IWP sensor. (**a) Dynamic response to NO_2_ concentrations (0–16 ppm) at room temperature; (b) Concentration-dependent sensitivity profile (0–16 ppm) with fitted curve; (c) Reproducibility demonstrated through five consecutive response cycles to 1 ppm NO_2_; (d) Selectivity assessment against various interfering gases; (e) Long-term stability evaluation for 1 ppm NO_2_ over 15 days of continuous operation.

**Figure S33** Gas responses of IWP sensor to mixed gases of (a) 1 ppm NO_2_ with varying NH_3_ concentrations(0, and 5 ppm NH_3_), and (b) 1 ppm NO_2_ with varying CO concentrations (0, and 100 ppm CO).

**Figure S34**. (a-c) Dynamic response curve of the IWP sensor to 1 ppm NO₂ at 80% RH. (d) Gas responds of the IWP sensor to 1 ppm NO_2_ gas at 30% and 80% RH.

**Figure S35. Reproducibility evaluation of the IWP sensors based on three independently fabricated devices.** (a–c) Response curves with error bars for (a) humidity sensing over relative humidity levels from 2%–98% RH, (b) pressure sensing over pressures ranging from 0.24–24 kPa, and (c) NO_2_ sensing over concentrations ranging from 0.2–16 ppm. Error bars represent the standard deviations obtained from three independently fabricated sensors.

**Figure S36. The infrared thermal imaging for the IWP sensor under continuous VNA monitoring.** It can be found that the power remains low enough to prevent heating issues on the skin even after 1 hour of continuous operation.

**Figure S37. Comparison of the sensing performance between single excitation and multiplexed readout modes.** (a) The resonance frequency shifts (Δ*f*) and (b) *S*_11_ gains of the humidity, pressure, and NO_2_ sensing units when interrogated individually (single excitation) versus simultaneously (multiplexed readout).

**Figure S38.** *S*_11_ signal loss of the humidity-, pressure-, and gas-sensing units in the IWP sensor before and after multiplexed readout.

**Figure S39. RF spectral responses to both individual and co-applied pressure and NO_2_ stimulus.** It indicates that applying pressure or introducing NO_2_ independently causes shifts in the resonance frequencies of the pressure or gas-sensing resonator units, respectively. When both stimuli are applied simultaneously, frequency shifts occur only in their respective resonators, with negligible change in the humidity unit.

**Figure S40. Photographs of the IWP sensor at various bending angles.** (a, b) 0°, (c, d) 15°, (e, f) 30°, (g, h) 45°, (i, j) 60°, and (k, l) 90°.

**Figure S41 Wireless transmission stability of the IWP sensor at various bending angles. (**a) The corresponding *S*_11_ reflection spectra measured at different bending angles. (b-d) The resonance frequency variations of the three LC resonator units as a function of the bending angle. As the bending angle increases, the effective electromagnetic coupling area decreases, leading to a gradual reduction in the overall *S*_11_ amplitude. Nevertheless, across all tested bending angles, the resonance frequencies of the sensor exhibit no obvious shifts. Quantitatively, the maximum angle coefficient of the baseline resonance frequency under bending is 0.0078 MHz/°. When translated into equivalent humidity, pressure, and gas perturbations, these variations correspond to only 0.0136 % RH/°, 0.0013 kPa/° and 0.0045 ppm/°, respectively. Such small equivalent stimulus offsets are negligible compared with the effective measurement ranges in practical applications. This demonstrates that the sensor maintains a stable baseline resonance frequency when subjected to bending deformations during wear.

**Figure S42. Photographs of the experimental setup for evaluating wireless transmission stability under dynamic tilting conditions.** The images display the IWP sensor tilted at various angles relative to the external readout coil: (a, b) -30°, (c, d) -20°, (e, f) -10°, (g, h) 0°, (i, j) 10°, (k, l) 20°, and (m, n) 30°.

**Figure S43. Wireless transmission stability of the IWP sensor at various tilting angles.** (a) The corresponding *S*_11_ reflection spectra measured at different tilting angles. (b-d) The resonance frequency variations of the three LC resonator units as a function of the tilting angle. The results show that non-parallel alignment alters the local magnetic coupling strength. Consequently, the *S*_11_ amplitude increases on the side closer to the readout coil and decreases on the farther side. Nevertheless, across all tested tilting angles, the resonance frequencies of the sensor exhibit no obvious shifts. Quantitatively, the maximum angle coefficient of the baseline resonance frequency under tilting is 0.0226 MHz/°. When translated into equivalent humidity, pressure, and gas perturbations, these variations correspond to only 0.0394 % RH/°, 0.0038 kPa/° and 0.0131 ppm/°, respectively. Such small equivalent stimulus offsets are negligible compared with the effective measurement ranges in practical applications. This indicates that the sensor maintains a stable baseline resonance frequency when subjected to different tilting angles.

**Figure S44. Wireless transmission stability of the IWP sensor within the physiological skin-temperature range of 32.2–38.4 °C.** (a-d) The *S*_11_ reflection spectra measured within the human skin temperature range (32.2-38.4 °C) [S29,S30], where (a) shows the overall spectra, and (b-d) present the enlarged views of the resonance peaks for the (b) humidity-sensing unit, (c) pressure-sensing unit, and (d) NO_2_ gas-sensing unit. (e-g) Extracted baseline resonance frequency variations (Δ*f*) and the corresponding linear fitting curves for the three sensing units as a function of temperature.

**Figure S45. Robustness of the decoupled multimodal sensing performance under environmental perturbations.** Resonance frequency shifts of the resonator array towards multimodal stimuli of 40% RH humidity, 4.8 kPa pressure, and 2 ppm NO_2_ under (a) 0° and 15° tilting (b) 20 °C and 40 °C temperature conditions. The results demonstrate that the functional sensitivities of the three modalities remain highly stable and distinguishable despite physical deformation and temperature variations, confirming that environmental drifts do not compromise the decoupling effect.

**Figure S46. Decoupled sensing performance between humidity and NO_2_ gas.** Simultaneous detection response for both NO_2_ (1.5 ppm) and varying humidity (20–60% RH) stimulus.

**Figure S47. Decoupled sensing performance toward simultaneous humidity and pressure.** Dynamic response curves showing resonance frequency variations under cyclic pressure (0.72 kPa) applied to the pressure-sensing unit and a gradual increase in humidity from 20% to 60% RH.

**Figure S48.** Synchronous resonance-frequency shifts of the (a) humidity-, (b) gas-, and (c) pressure-sensing units as a function of simultaneously applied humidity levels (20%, 40%, and 60% RH), NO_2_ concentrations (1, 2, and 8 ppm), and cyclic gradient pressures (0.24, 0.72, and 1.7 kPa).

**5. Supplementary References**

S1. T. Y. Kim, K. Son, C.-H. Moon, et al., “Quinone-mediated, tissue-adaptive double-network hydrogel for instant hemostasis and wet-tissue adhesion,” *Nature Communications* 17 (2026): 5526, https://doi.org/10.1038/s41467-026-72068-6.

S2. D. M. Pozar 2012, "Microwave engineering." in Fourth Editions, University of Massachusetts at Amherst, John Wiley & Sons, Inc. ISBN 978-0-470-63155-3.

S3. Q. Yu, S. Jin, S. Wang, H. Xiao, Y. Zhao, “Injectable, adhesive, self-healing and conductive hydrogels based on MXene nanosheets for spinal cord injury repair,” *Chemical Engineering Journal* 452 (2023): 139252, https://doi.org/10.1016/j.cej.2022.139252.

S4. B. Dober, D. T. Becker, D. A. Bennett, et al., “Microwave SQUID multiplexer demonstration for cosmic microwave background imagers,” *Applied Physics Letters* 111 (2017): 243510, https://doi.org/10.1063/1.5008527.

S5. J. A. B. Mates, D. T. Becker, D. A. Bennett, et al., “Crosstalk in microwave SQUID multiplexers,” *Applied Physics Letters* 115 (2019): 202601, https://doi.org/10.1063/1.5116573.

S6. C. A. Balanis 2016, "Antenna theory: analysis and design." John wiley & sons. ISBN 1118642066.

S7. X. Huang, Y. Liu, H. Cheng, et al., “Materials and Designs for Wireless Epidermal Sensors of Hydration and Strain,” *Advanced Functional Materials* 24 (2014): 3846–3854, https://doi.org/10.1002/adfm.201303886.

S8. Q.-A. Huang, L. Dong, L.-F. Wang, “LC Passive Wireless Sensors Toward a Wireless Sensing Platform: Status, Prospects, and Challenges,” *Journal of Microelectromechanical Systems* 25 (2016): 822–841, https://doi.org/10.1109/jmems.2016.2602298.

S9. H. Miao, J. Bi, Y. Gao, et al., “A microstructure‐enhanced dual‐mode LC sensor with a PSO‐BP algorithm for precise detection of temperature and pressure,” *Advanced Functional Materials* 34 (2024): 2408198, https://doi.org/10.1002/adfm.202408198.

S10. P. Wang, G. Q. Wang, G. F. Sun, et al., “A Flexible-Integrated Multimodal Hydrogel-Based Sensing Patch,” *Nano-Micro Letters* 17 (2025): 156, https://doi.org/10.1007/s40820-025-01656-w.

S11. H. Zhang, H. Chen, J. H. Lee, et al., “Bioinspired Chromotropic Ionic Skin with In‐Plane Strain/Temperature/Pressure Multimodal Sensing and Ultrahigh Stimuli Discriminability,” *Advanced Functional Materials* 32 (2022): 2208362, https://doi.org/10.1002/adfm.202208362.

S12. Y. H. Liu, J. L. Wang, T. Liu, et al., “Triboelectric tactile sensor for pressure and temperature sensing in high-temperature applications,” *Nature Communications* 16 (2025): 383, https://doi.org/10.1038/s41467-024-55771-0.

S13. X. Lyu, H. Zhang, S. Shen, Y. Gong, P. Zhou, Z. Zou, “Multi‐Modal Sensing Ionogels with Tunable Mechanical Properties and Environmental Stability for Aquatic and Atmospheric Environments,” *Advanced Materials* 36 (2024): 2410572, https://doi.org/10.1002/adma.202410572.

S14. D. Lee, J. Bae, “Soft Multimodal Sensors with Decoupled Multimodality and Minimal Wiring for Wearable Systems,” *Advanced Functional Materials* 34 (2024): 2409841, https://doi.org/10.1002/adfm.202409841.

S15. L. Luo, Z. Wu, Q. Ding, et al., “In Situ Structural Densification of Hydrogel Network and Its Interface with Electrodes for High-Performance Multimodal Artificial Skin,” *ACS Nano* 18 (2024): 15754–15768, https://doi.org/10.1021/acsnano.4c02359.

S16. W. Wang, D. Yao, H. Wang, et al., “A Breathable, Stretchable, and Self‐Calibrated Multimodal Electronic Skin Based on Hydrogel Microstructures for Wireless Wearables,” *Advanced Functional Materials* 34 (2024): 2316339, https://doi.org/10.1002/adfm.202316339.

S17. Y. Yang, L. Kong, B. Huang, B. Lin, L. Fu, C. Xu, “A High‐Sensitive Rubber‐Based Sensor with Integrated Strain and Humidity Responses Enabled by Bionic Gradient Structure,” *Advanced Functional Materials* 34 (2024): 2400789, https://doi.org/10.1002/adfm.202400789.

S18. C. Yang, H. Wang, J. Yang, et al., “A Machine-Learning-Enhanced Simultaneous and Multimodal Sensor Based on Moist-Electric Powered Graphene Oxide,” *Advanced Materials* 34 (2022): 2205249, https://doi.org/10.1002/adma.202205249.

S19. S. Sharma, G. B. Pradhan, S. Jeong, S. Zhang, H. Song, J. Y. Park, “Stretchable and All-Directional Strain-Insensitive Electronic Glove for Robotic Skins and Human–Machine Interfacing,” *ACS Nano* 17 (2023): 8355–8366, https://doi.org/10.1021/acsnano.2c12784.

S20. X. Zhang, S. Tang, R. Ma, et al., “High-performance multimodal smart textile for artificial sensation and health monitoring,” *Nano Energy* 103 (2022): 107778, https://doi.org/10.1016/j.nanoen.2022.107778.

S21. L. Chen, X. Chang, H. Wang, J. Chen, Y. Zhu, “Stretchable and transparent multimodal electronic-skin sensors in detecting strain, temperature, and humidity,” *Nano Energy* 96 (2022): 107077, https://doi.org/10.1016/j.nanoen.2022.107077.

S22. Q. Tan, W. Lv, Y. Ji, et al., “A LC wireless passive temperature-pressure-humidity (TPH) sensor integrated on LTCC ceramic for harsh monitoring,” *Sensors and Actuators B-Chemical* 270 (2018): 433–442, https://doi.org/10.1016/j.snb.2018.04.094.

S23. L. Dong, L.-F. Wang, Q.-A. Huang, “An *LC* Passive Wireless Multifunctional Sensor Using a Relay Switch,” *IEEE Sensors Journal* 16 (2016): 4968–4973, https://doi.org/10.1109/jsen.2016.2550537.

S24. H. Kou, Q. Tan, L. Zhang, H. Dong, J. Xiong, W. Zhang, “Highly Sensitive Air-Filled Substrate Integrated Waveguide Resonator Integrated Wireless Passive Slot-Antenna for Confined Environmental Detection,” *IEEE Sensors Journal* 19 (2019): 10027–10033, https://doi.org/10.1109/jsen.2019.2921148.

S25. C. Liu, R. Zhang, Y. Wang, et al., “Tough, anti-drying and thermoplastic hydrogels consisting of biofriendly resources for a wide linear range and fast response strain sensor,” *Journal of Materials Chemistry A* 11 (2023): 2002–2013, https://doi.org/10.1039/d2ta06822b.

S26. M. D. Figueroa-Pizano, I. Velaz, F. J. Penas, et al., “Effect of freeze-thawing conditions for preparation of chitosan-poly (vinyl alcohol) hydrogels and drug release studies,” *Carbohydrate Polymers* 195 (2018): 476–485, https://doi.org/10.1016/j.carbpol.2018.05.004.

S27. Y. Wei, L. Xiang, H. Ou, et al., “MXene-Based Conductive Organohydrogels with Long-Term Environmental Stability and Multifunctionality,” *Advanced Functional Materials* 30 (2020): 2005135, https://doi.org/10.1002/adfm.202005135.

S28. Z.-J. Chen, T.-Y. Shen, M.-H. Zhang, et al., “Tough, Anti-Fatigue, Self-Adhesive, and Anti-Freezing Hydrogel Electrolytes for Dendrite-Free Flexible Zinc Ion Batteries and Strain Sensors,” *Advanced Functional Materials* 34 (2024): 2314864, https://doi.org/10.1002/adfm.202314864.

S29. P. Webb, “Temperatures of skin, subcutaneous tissue, muscle and core in resting men in cold, comfortable and hot conditions,” *European Journal of Applied Physiology and Occupational Physiology* 64 (1992): 471–476, https://doi.org/10.1007/bf00625070.

S30. W. Bierman, “The temperature of the skin surface,” *Journal of the American Medical Association* 106 (1936): 1158–1162, https://doi.org/10.1001/jama.1936.02770140020007.
